# Combined metabolome and transcriptome profiling provides new insights into diterpene biosynthesis in *S. pomifera* glandular trichomes

**DOI:** 10.1186/s12864-015-2147-3

**Published:** 2015-11-14

**Authors:** Fotini A. Trikka, Alexandros Nikolaidis, Codruta Ignea, Aphrodite Tsaballa, Leto-Aikaterini Tziveleka, Efstathia Ioannou, Vassilios Roussis, Eleni A. Stea, Dragana Božić, Anagnostis Argiriou, Angelos K. Kanellis, Sotirios C. Kampranis, Antonios M. Makris

**Affiliations:** Institute of Applied Biosciences/CERTH, P.O. Box 60361, Thermi, 57001, Thessaloniki Greece; Department of Biochemistry, School of Medicine, University of Crete, P.O. Box 2208, Heraklion, 71003 Greece; Department of Pharmacognosy and Chemistry of Natural Products, Faculty of Pharmacy, University of Athens, Panepistimiopolis Zografou, Athens, 15771 Greece; Group of Biotechnology of Pharmaceutical Plants, Laboratory of Pharmacognosy, Department of Pharmaceutical Sciences, Aristotle University of Thessaloniki, 541 24 Thessaloniki, Greece; Institute for Biological Research “Siniša Stanković”, University of Belgrade, Belgrade, Serbia

**Keywords:** Miltiradiene, *S. pomifera*, Diterpenes, Cytochrome P450

## Abstract

**Background:**

*Salvia* diterpenes have been found to have health promoting properties. Among them, carnosic acid and carnosol, tanshinones and sclareol are well known for their cardiovascular, antitumor, antiinflammatory and antioxidant activities. However, many of these compounds are not available at a constant supply and developing biotechnological methods for their production could provide a sustainable alternative. The transcriptome of *S.pomifera* glandular trichomes was analysed aiming to identify genes that could be used in the engineering of synthetic microbial systems.

**Results:**

In the present study, a thorough metabolite analysis of *S. pomifera* leaves led to the isolation and structure elucidation of carnosic acid-family metabolites including one new natural product. These labdane diterpenes seem to be synthesized through miltiradiene and ferruginol. Transcriptomic analysis of the glandular trichomes from the *S. pomifera* leaves revealed two genes likely involved in miltiradiene synthesis. Their products were identified and the corresponding enzymes were characterized as copalyl diphosphate synthase (SpCDS) and miltiradiene synthase (SpMilS). In addition, several CYP-encoding transcripts were identified providing a valuable resource for the identification of the biosynthetic mechanism responsible for the production of carnosic acid-family metabolites in *S. pomifera*.

**Conclusions:**

Our work has uncovered the key enzymes involved in miltiradiene biosynthesis in *S. pomifera* leaf glandular trichomes. The transcriptomic dataset obtained provides a valuable tool for the identification of the CYPs involved in the synthesis of carnosic acid-family metabolites.

**Electronic supplementary material:**

The online version of this article (doi:10.1186/s12864-015-2147-3) contains supplementary material, which is available to authorized users.

## Background

*Salvia* species have attracted great attention due to their biologically active constituents [1]. Two major groups of secondary metabolites are produced in *Salvia* species; terpenoids and polyphenolics. Diterpenes is the largest group comprising 545 of 791 presently identified *Salvia* sp. constituents, with labdanes being the main metabolites [1]. Carnosol and carnosic acid, two diterpenes naturally found in sage and rosemary, have been evaluated for their anti-inflammatory [2], antioxidant [3, 4] and anticancer properties [5, 6]. Tanshinones, naturally found in *S. miltiorrhiza* roots, are widely used in the treatment of cardiovascular diseases [7–9]. They are a mix of chemical compounds mainly consisting of tanshinone I, tanshinone IIA, cryptotanshinone and dihydrotanshinone having various biological activities, such as anti-inflammatory [10], antibacterial [11], antioxidant [12] and antineoplastic [13–15]. So far, extracts of *S. miltiorrhiza* roots have been successfully enrolled in clinical trials for coronary heart disease [16], pulmonary hypertension [17] and polycystic ovary syndrome [18].

In plants, terpenes are synthesized via two pathways: the mevalonate pathway in the cytosol and the 2-C-methyl-D- erythritol-4-phosphate (MEP) pathway in the plastids. The main building block of terpenes is an isoprene unit that is derived from isopentenyl diphosphate (IPP) and dimethylallyl diphosphate (DMAPP). Then, by the action of prenyltransferases on IPP and DMAPP, the higher building blocks of terpenes are generated: geranyl diphosphate (GPP) for monoterpene synthesis, (*E, E*)-farnesyl diphosphate (FPP) for sesquiterpene and (*E, E, E*)-geranylgeranyl diphosphate (GGPP) for diterpene synthesis [19]. Diterpenes are mainly synthesized via the MEP pathway; nevertheless a crosstalk between the pathways has been reported [20].

Tanshinones and carnosic acid, derive from the universal diterpenoid precursor GGPP that is subsequently converted to copalyl diphosphate (CDP) by the action of CDP synthase. Additional cyclization catalyzed by a class I terpene synthase (SmKSL) results in the formation of miltiradiene [21]. Subsequently, the action of cytochrome P450 monooxygenases, such as CYP76AH1 from *S. miltiorrhiza* [22] or CYP76AH4 from *R. officinalis* [23], catalyzes the synthesis of ferruginol, a potential precursor of carnosic acid which in turn may be an intermediate to tanshinone biosynthesis [22]. Due to the biological significance of carnosol, carnosic acid and tanshinones, there is increasing need for improved or alternative methods of production of these metabolites. As a result, efforts aiming to elucidate the related biosynthetic pathways has led to the identification of several enzymatic steps, not only in *S. miltiorrhiza* [24, 25] but also in other species [26, 27]. Specifically, diterpene synthases catalyzing the same enzymatic reactions with SmCDS and SmKSL have been identified in *Rosmarinus officinalis* [26] and in *Salvia fruticosa* [27]. Identification of ferruginol synthase in *S. miltiorrhiza*, *R. officinalis* and *S. fruticosa* enabled the synthesis of ferruginol in yeast cells [22, 27]. Aiming to reconstruct tanshinone biosynthesis in yeast, expression of *S. miltiorrhiza* ferruginol synthase CYP76AH1 in a dedicated strain resulted in 10.5 mg/L of ferruginol [22]*.*

In the current study, we conducted a combined metabolome and transcriptome analysis of *S. pomifera* leaves and leaf trichomes, respectively, in order to provide an insight into isoprenoid pathway and identify key enzymes involved in terpene biosynthesis. Metabolite analysis of *S. pomifera* leaf extracts led to the identification and isolation of several carnosic acid-related metabolites, suggested that transcriptomic analysis may yield important insights into the biosynthesis of these compounds. Two genes that appeared to be likely involved in miltiradiene synthesis, a copalyl diphosphate synthase (SpCDS) and a miltiradiene synthase (SpMilS) were cloned and expressed in *S. cerevisiae* and their products were characterized. Furthermore, from the full transcriptomic profile of *S. pomifera’s,* we identified the CYPs providing a useful insight on the subsequent biosynthetic steps.

## Results

### Isolation and characterization of diterpenes from *S.pomifera* leaves

**Fig. 1:**
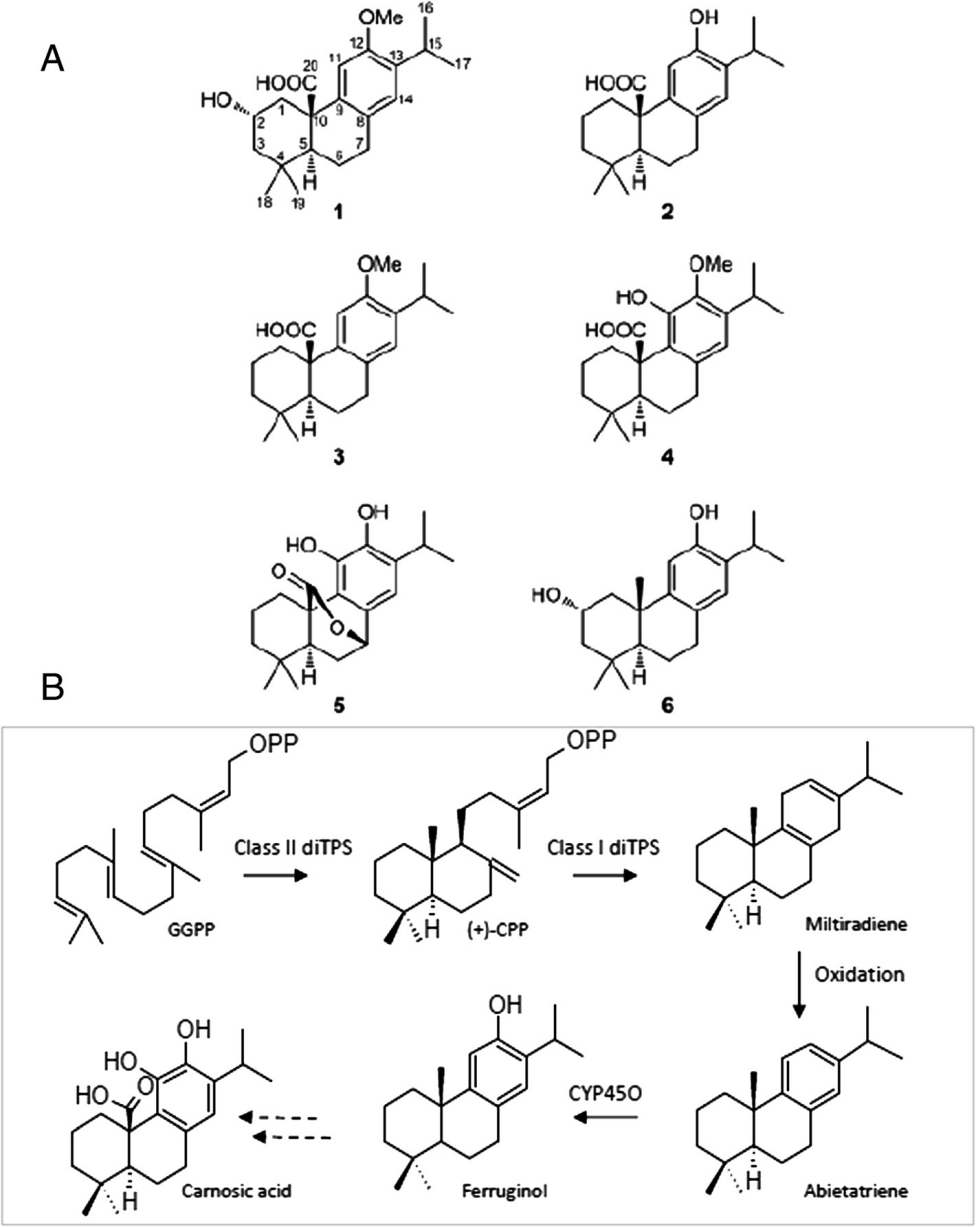
Structures and biosynthesis of the main diterpenes found in *S. pomifera* leaves. **a** The names of the isolated compounds from *S. pomifera* leaves are: (**1**) 2*α*-hydroxy-*O*-methyl-pisiferic acid, (**2**) pisiferic acid, (**3**) *O*-methyl-pisiferic acid, (**4**) 12-methoxycarnosic acid, (**5**) carnosol and (**6**) salviol. **b** The proposed biosynthetic pathway of carnosic acid-family metabolites: Geranylgeranyl diphosphate (GGPP) is subjected to cyclization by a class II diterpene synthase to form copalyl diphosphate ((+)-CPP). Then, by the action of a class I diterpene synthase, miltiradiene is formed. Subsequent oxidation and cytochrome P450 activity results in the formation of ferruginol. Figure was produced using Chemescketch v 14.

Specimens of *S. pomifera* from the areas of Topolia (Western Crete, Greece) and Aptera (North-western Crete, Greece) were collected and the constituents of their aerial parts were extracted. A series of chromatographic separations of the organic extracts resulted in the isolation of one new (**1**) and five previously reported metabolites, which were identified as pisiferic acid (**2**), *O*-methyl-pisiferic acid (**3**), 12-methoxycarnosic acid (**4**), carnosol (**5**), and salviol (**6**) (Fig. 1a), by comparison of their spectroscopic and physical characteristics with those reported in the literature [28–33].

The molecular formula of compound **1**, isolated as a white solid, was determined to be C_21_H_30_O_4_ on the basis of its HR-ESI-MS data. The spectroscopic characteristics of metabolite **1** were rather similar to those of compounds **2**–**6**, indicating an abietane skeleton. Its ^1^H NMR spectrum included signals for two aromatic protons resonating at *δ* 6.72 and 6.91, one oxygenated methine at *δ* 4.26, one oxymethyl at *δ* 3.73, two singlet methyls at *δ* 1.00 and 0.82 and two doublet methyls at *δ* 1.16 and 1.14 (Table 1). Analysis of the correlations observed in the HSQC, COSY and HMBC spectra led to the complete assignment of the protons and carbons of the molecule, while the relative configuration was proposed on the basis of NOE enhancements. In particular, the heteronuclear correlations of C-20 with H-1*α* and H-5 indicated the position of the carboxylic acid at C-20, while the correlations of C-12 with the protons of the oxymethyl and H-15 indicated the position of the methoxy group at C-12. Furthermore, the heteronuclear correlations of C-8 and C-13 with H-11 and of C-9 and C-12 with H-14 indicated the position of the aromatic protons. The NOE interactions of H-11 with the protons of the oxymethyl and H-1*α* and of H-14 with H_2_-7, H_3_-16 and H_3_-17 further confirmed the position of the aromatic protons. Additionally, the NOE interactions of H-2 with H-1*β*, H-3*β* and H_3_-19, in combination with the coupling constants of H-2 determined the equatorial orientation of the hydroxyl group at C-2. Thus, compound **1** was identified as 2*α*-hydroxy-*O*-methyl-pisiferic acid. The ^1^H-NMR and ^13^C-NMR spectra of 2*α*-hydroxy-*O*-methyl-pisiferic acid are provided as Additional file 1: Figure S2 and Additional file 2: Figure S3 along with the 1H-NMR spectra of pisiferic acid (Additional file 3: Figure S4), *O*-methyl-pisiferic acid (Additional file 4: Figure S5), 12-methoxycarnosic acid (Additional file 5: Figure S6), carnosol (Additional file 6: Figure S7) and salviol (Additional file 7: Figure S8).

**Table 1:** ^1^H and ^13^C NMR data (in CDCl_3_) of 2*α*-hydroxy-*O*-methyl-pisiferic acid (compound 1)

	*δ*_C_	*δ*_H_ (*J* in Hz)
1α	45.2	1.24, m
1β		3.14, m
2α	65.4	4.26, tt (11.5, 4.4)
3α	50.5	1.24, m
3β		1.82, m
4	34.9	
5	51.6	1.50, dd (13.0, 2.7)
6α	18.2	1.89, m
6β		2.42, dddd (13.0, 13.0, 10.8, 6.6)
7α	29.1	2.80, ddd (17.0, 10.8, 7.5)
7β		2.92, dd (17.0, 6.6)
8	128.6	
9	136.8	
10	48.5	
11	107.1	6.72, s
12	155.0	
13	136.3	
14	127.2	6.91, s
15	26.5	3.19, sept (6.8)
16	22.4	1.14, d (6.8)
17	22.8	1.16, d (6.8)
18	32.1	1.00, s
19	20.9	0.82, s
20	179.9	
OCH_3_	55.4	3.73, s

These structures of carnosic acid- family metabolites seem to be derived from ferruginol via miltiradiene and abietatriene (Fig. 1b). The presence of small amounts of ferruginol in extracts of the aerial parts of *S. pomifera* (data not shown) supports this hypothesis. In order to elucidate the biosynthesis of these compounds, we proceeded with the analysis of the transcriptome of *S. pomifera* glandular trichomes.

### Transcriptome sequencing, *de novo* assembly and sequence clustering of *S. pomifera* trichome transcripts

Sequencing of *S. pomifera* leaf trichomes resulted in approximately 54,000,000 raw reads from which nearly 85 % were of high quality. The results of the RNA-Seq regarding the throughput and the quality of *S. pomifera* leaf’s trichome libraries are included in Table 2. High quality reads were assembled into contigs using the standard pipeline of the Trinity suite software [34]. A K-mer catalogue was produced by jellyfish software and used as input for inchworm to assemble the contigs. Subsequently, reads were mapped back to the assembled contigs to improve assembly using bowtie. In total, 125,371 contigs were assembled with a mean length of 321 nt (Additional file 8: Figure S1A). These contigs were re-assembled into 66,051 larger contigs of 640 nt mean length; however the majority of the contigs was less than 600 nt in length (Additional file 8: Figure S1B). From these, 22,395 were clustered in clusters of contigs (given the prefix cl) and 43,656 remained as singletons (given the prefix unigene) in the final Trinity output produced by the coordinated action of Chrysalis and Butterfly software. Contigs with less than 600 nt in length had in average 600 total reads mapped in each contig.

**Table 2:** An overview of the RNA-seq outcome including numbers for total raw, high quality reads and nucleotides and the statistical Q20, N and GC percentages

	Total raw reads	Total high quality reads	Total clean nucleotides	Q20 (%)	N (%)	GC (%)
*S. pomifera* transcriptome	54.00 M	45.89 M	4.13 G	95.60	0.00	49.40

### Functional annotation and transcript expression in trichomes

*S. pomifera* contigs were employed in BLAST searches against several biological databases for their annotation. 70 % of the *S. pomifera* contigs had a non-redundant (nr) NCBI BLAST hit, while the percentage of the sequences that were annotated based on Gene Ontology (GO) was 50.4 %. The percentages of the sequences annotated based on BLAST searches against the Nucleotide database (Nt), Swiss-Prot and Kyoto Encyclopedia of Genes and Genomes (KEGG), were similar; 49.74, 44.5 and 41.9 %, respectively. Much lower was the percentage of *S. pomifera* contigs that were assigned to Clusters of Orthologous Groups (COG), 26.2 %.

Most of the contigs that had a hit against the nr protein database shared significant similarity, 37 %, with *Vitis vinifera* sequences, 13 % were similar to *Ricinus communis* sequences, 11.4 % to *Populus trichocarpa*, 8 % to *Glycine max* and 23.8 % to other plants. The majority of the contigs, 40.4 %, showed a similarity level of 60–80 % with proteins from the nr database and 21 % of the contigs had a similarity with proteins that extended beyond 80 %.

**Fig. 2:**
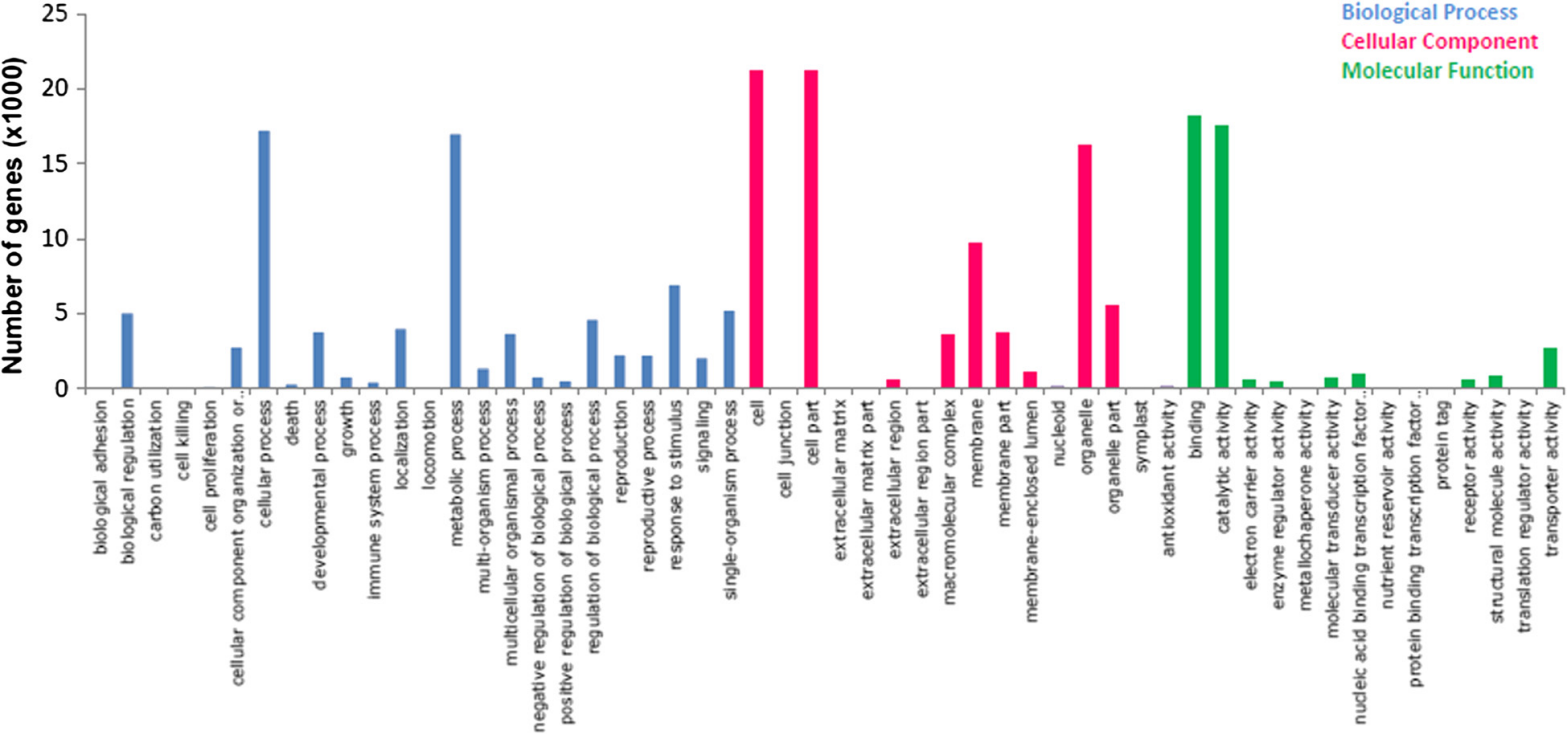
The GO annotation of *S. pomifera* leaf trichomes contigs. Assignment of *S. pomifera* contigs on Gene Ontologies: biological process, cellular component and molecular function and their sub-categories

Among the 33,332 transcripts with at least one GO-term assigned, the highest percentage of transcripts in the cellular component ontology were annotated in the cell/cell part class (21,126) in the molecular function ontology the majority of the transcripts were in binding (18,119) while in the biological process ontology most of the transcripts were grouped in the cellular process class (17,210). The detailed classification of the unigenes in the individual GO-terms of the three GO ontology domains is depicted in Fig. 2. Similar results were obtained from the GO-analysis of transcripts sequenced from glandular and non-glandular tomato stem trichomes [35].

**Fig. 3:**
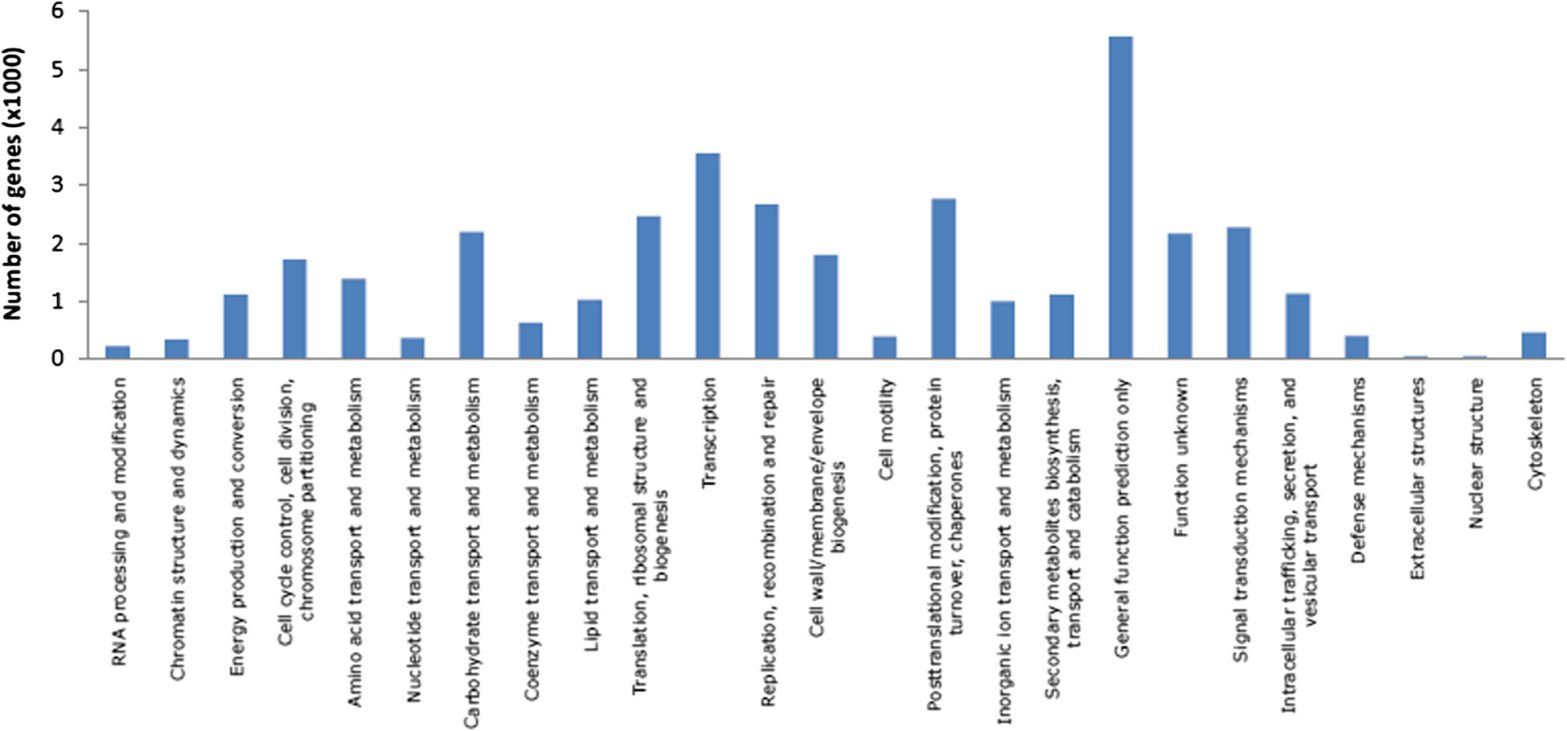
Classification of *S. pomifera* transcripts in the 25 COG categories

Furthermore, 17,303 *S. pomifera* transcripts were classified into 25 COG functional categories. For most of the transcripts only a general function prediction was made while the next two most abundant categories were transcription and post-translational modification, protein turnover and chaperones. Extracellular and nuclear structures are the two COG categories with the smallest number of assigned transcripts. The complete classification of *S. pomifera* transcripts according to COG is depicted in Fig. 3. The identity of the most expressed transcripts in *S. pomifera* leaf trichomes, their top nr BLAST hits, and their GO annotation are presented in Table 3.

**Table 3:** The 35 most expressed transcripts in *S. pomifera* leaf trichomes, along with their length, expression in trichomes (in FPKM values), top BLAST NR hit, % identity with the top hit and its GO annotation

*S. pomifera* transcript	Length	FPKM	Top BLAST NR hit	% identity	GO annotation
Unigene34508	248	30,475.2	Hypothetical protein NitaMp027 [TOBAC]	99	GO:0005739
Unigene5960	726	11,636.4	No hit		
CL3171.Contig2	980	10,338.5	Predicted: ribulose bisphosphate carboxylase small chain, chloroplastic-like [EUCGR]	75	GO:0019253
CL2955.Contig2	677	8953.6	No hit		
CL8497.Contig1	706	7747.5	Lipid transfer protein [SESIN]	81	GO:0008289
Unigene34505	997	6766.4	Predicted: uncharacterized protein LOC105170030 [SESIN]	66	GO:0044464
CL9261.Contig1	1266	6323.3	Predicted: GDSL esterase/lipase At5g33370-like [SESIN]	78	GO:0006629
Unigene33614	1136	5655.3	SMLII [SALMI]	72	
CL3502.Contig4	560	5539.9	Lipid transfer protein 2 [SALMI]	93	GO:0006810
Unigene10249	266	5028.6	Lipid transfer protein 1 [SALMI]	85	
Unigene34506	679	4980.5	No hit		
CL2203.Contig2	913	4616.1	Pathogenesis-related protein 10 [SALMI]	85	GO:0009607
CL3171.Contig3	831	4219.9	Predicted: ribulose bisphosphate carboxylase small chain, chloroplastic-like [EUCGR]	75	GO:0019253
CL7758.Contig2	672	4140.8	Chlorophyll a/b-binding protein, partial [*Schiedea haleakalensis*]	97	GO:0046872
CL4643.Contig2	332	4047.4	Predicted: photosystem II 10 kDa polypeptide, chloroplastic [SESIN]	85	GO:0015979
CL1963.Contig2	569	3695.1	No hit		
Unigene19442	829	3596.8	Putative metallothionin 2a [SALMI]	95	GO:0046872
Unigene5947	858	3446.2	Histone H3.3 [ARATH]	100	GO:0000786
Unigene31727	274	3056.0	BnaC06g21170D [BRANA]	91	GO:0016023
CL5332.Contig1	791	2943.3	No hit		
CL1258.Contig1	1538	2773.7	Chloroplast ribulose-1,5-bisphosphate carboxylase/oxygenase activase [PLESU]	87	GO:0005524
CL495.Contig1	2029	2742.6	Predicted: uncharacterized protein LOC104647985 [SOLLC]	54	
CL1454.Contig2	1262	2666.7	Predicted: dehydration-responsive protein RD22 [SESIN]	62	
CL2498.Contig2	763	2658.0	Predicted: probable thionin-2.4 [CAMSA]	68	GO:0006952
Unigene22580	532	2506.2	Predicted: chlorophyll a-b binding protein, chloroplastic [NICTO]	93	GO:0046872
Unigene29603	788	2494.0	Non-specific lipid-transfer protein 3 [ARATH]	50	GO:0009737
Unigene34507	653	2393.6	Predicted: photosystem I reaction center subunit VI, chloroplastic-like [SESIN]	93	GO:0010287
CL2671.Contig2	511	2357.7	Predicted: probable protein Pop3 [SESIN]	49	
Unigene32013	334	2357.0	Chloroplast photosystem II 10 kDa protein, partial [ARAHY]	94	GO:0015979
Unigene10346	336	2309.4	1-hydroxy-2-methyl-butenyl 4-diphosphate reductase [CATRO]	43	
CL182.Contig3	3830	2269.2	Sabinene synthase, partial [*S. pomifera*]	100	GO:0005525
CL9337.Contig1	2282	2183.6	Cinnamoyl CoA reductase [SALMI]	93	GO:0009809
Unigene10272	1075	2181.4	Predicted: photosystem I reaction center subunit XI, chloroplastic [SESIN]	93	GO:0010287
Unigene34510	622	2160.2	Phytosulfokine precursor [AVIMR]	62	GO:0007275
Unigene32188	693	2132.8	Predicted: chlorophyll a-b binding protein 1D-like [NELNU]	96	GO:0046872

Most of the *S. pomifera* contigs that are strongly expressed in leaf trichomes share significant similarity with annotated nr sequences. Among them, two abundantly expressed *S. pomifera* contigs are involved in secondary metabolism; cl182.contig3 (3830 nt) encoding the previously characterized sabinene synthase [36] with one amino acid difference, and cl.contig1 (2282 nt) coding for a full length protein that is highly similar to cinnamoyl CoA reductase (GenBank: AFE84656.1). Apart from the annotated transcripts, unigene5960, cl2955.contig2, unigene34506, cl1963.contig2 and cl5332.contig1 produced no hits versus nr NCBI database and they were used in further BLAST searches in the European Nucleotide Archive (ENA) database. Unigene 5960 (length 726 nt) is highly similar to mRNA sequence FE536539 extracted from leaf trichomes of another Greek *Salvia* species, *S. fruticosa*. Transcript cl2955.contig2 (677 nt) has only one nucleotide change to mRNA sequence FE536032.1 from leaf trichomes of *S. fruticosa* cl5332. contig1 (791 nt) blasts against another *S. fruticosa* leaf trichome cDNA clone of ENA database (FE536766.1) with almost perfect identity. All the above sequences lack annotation which was also observed for a large number of transcripts in the trichomes of *Mentha spicata* [37].

### Expression analysis of genes involved in the isoprenoid biosynthesis

**Fig. 4:**
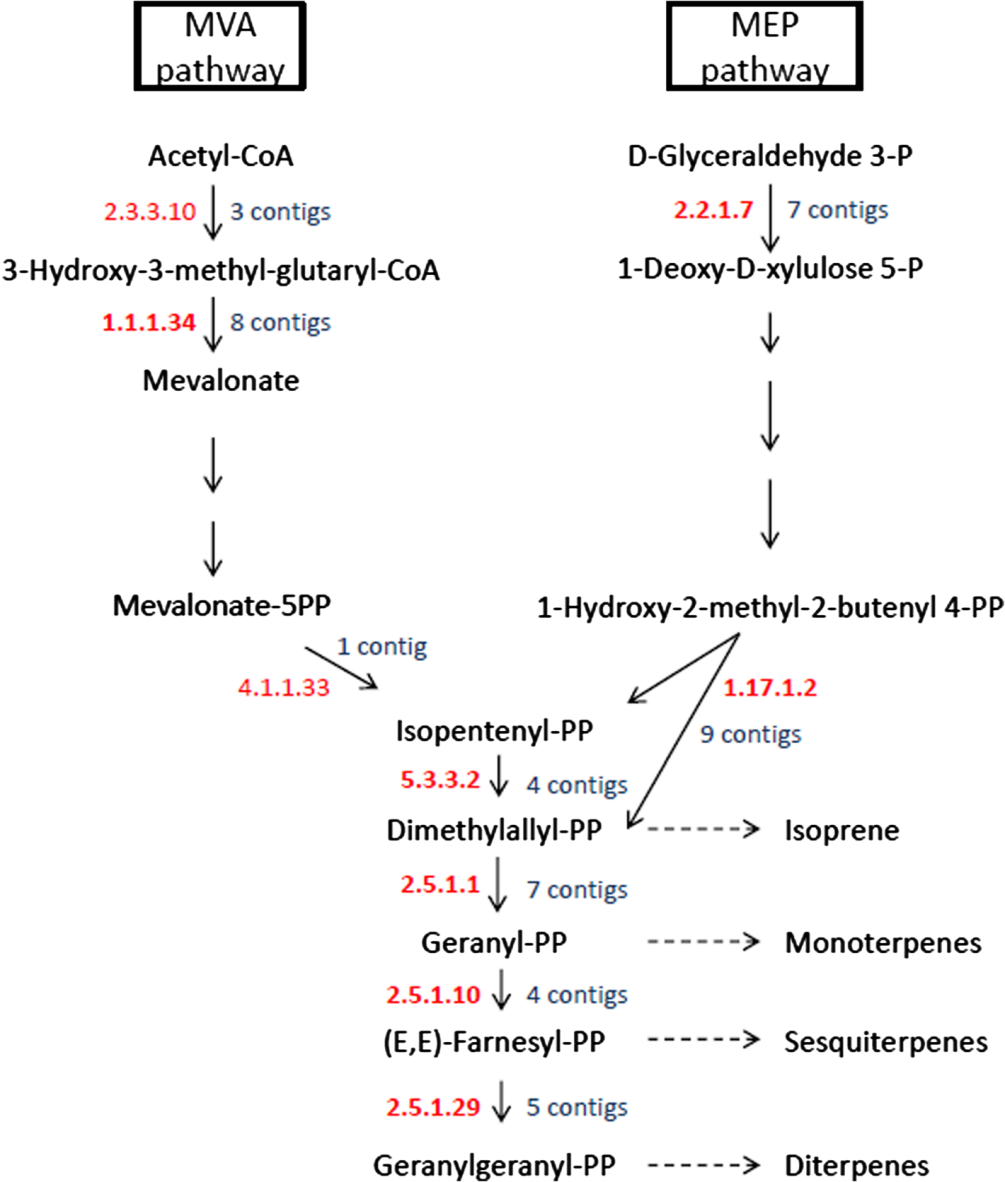
Graphical representation of the two pathways, MVA and MEP, by which plant terpenes are produced. The KEGG entries of the specific enzymes that participate in the pathways are shown in red as alongside the number of contigs identified that putatively encode for these enzymes

Several *S. pomifera* contigs were predicted to code for proteins that participate in the terpenoid precursor biosynthesis pathway (KEGG entry 00900). Terpenoid biosynthesis in plants is carried out via two pathways: the mevalonate pathway (MVA) in the cytosol and the non-mevalonate or 2-C-methyl-D-erythritol 4-phosphate/1-deoxy-D-xylulose 5-phosphate pathway (MEP/DOXP pathway) in the plastids. In Fig. 4, the two terpenoid biosynthetic pathways are depicted with their identified corresponding enzymes and the number of contigs that putatively code for these enzymes in the present transcriptomic analysis. The first protein/enzyme of the plastid MEP pathway, 1-deoxy-D-xylulose-5-phosphate synthase (DXS, EC: 2.2.1.7) is encoded by seven contigs; 6 contigs that contain a partial and 1 contig with a full ORF. Another important enzyme of the pathway, the 4-hydroxy-3-methylbut-2-enyl diphosphate reductase (HDR, EC: 1.17.1.2) is encoded by 9 *S. pomifera* contigs. On the other hand, enzyme 3-hydroxy-3-methylglutaryl-CoA reductase (HMGR, EC: 1.1.1.34) of the MVA pathway is encoded by 8 contigs.

**Fig. 5:**
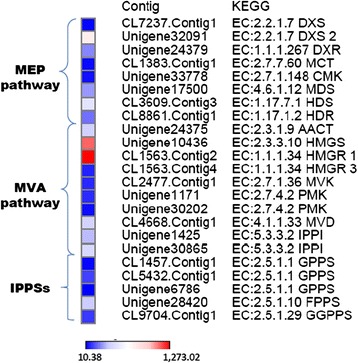
Expression heatmap of *S. pomifera* contigs. Contigs that encode full or almost full length proteins and are putatively involved in the MEP and MVA pathways of the terpenoid backbone biosynthesis in the leaf trichomes. Color bar at the bottom of the figure denotes color intensity according to global expression levels. Abbreviations; DXS: 1-deoxy-D-xylulose-5-phosphate synthase, DXR: 1-deoxy-D-xylulose-5-phosphate reductoisomerase, MCT: MEP cytidyltransferase, CMK: 4-(cytidine 5- diphospho)-2-C-methyl-D-erythritol kinase, MDS: 2-C-methyl-D-erythritol 2,4-cyclodiphosphate, HDS: 1-hydroxy-2-methyl-2-butenyl 4-diphosphate synthase, HDR: 1-hydroxy-2-methyl-2-butenyl 4-diphosphate reductase, HMG: 3-hydroxy-3-methylglutaryl, MVK: mevalonate kinase, PMK: phosphomevalonate kinase, MVD: diphosphomevalonate decarboxylase, IPPI: isopentenyl diphosphate, IPPSs: isoprenyl pyrophosphate synthases

The expression levels of the full or nearly full *S. pomifera* ORFs involved in terpenoid biosynthesis are presented in Fig. 5. The most expressed transcript of the MEP pathway is unigene32091 (2054 nt) which encodes for a DXS protein and shares high similarity (>90 %) with DXS2 from *S. miltiorrhiza* (GenBank: ACQ66107.1). The FPKM value for unigene32091 - putative *DXS2* is 688.42. In the MVA pathway, the highest expressed transcript is cl1563.contig2 (2263 nt) that encodes for a putative HMGR protein and has 87 % similarity with HMGR1 from *S. miltiorrhiza* (GenBank: ADC44451.1). The FPKM value for cl1563.contig2 - putative *HMGR1* is 1273.02, nearly double than that of unigene32091 of the MEP pathway. The second most abundant is unigene10436 (1978 nt) - FPKM value is 985.72 - encodes for a HMGS protein almost identical to HMGS1 from *S. miltiorrhiza* (GenBank: ACV65039.1). In general, the transcripts that are predicted to participate in the MEP pathway are present at lower levels than the transcripts of the MVA pathway. Finally, 16 *S. pomifera* contigs are putative GPP, GGPP and FPP synthases. Only one full length putative *GGPPS* gene, cl9704.contig1, is included in Fig. 5 with a rather moderate expression. Α putative *FPPS* gene i.e., unigene28420 that synthesizes FPP, the precursor of sesquiterpenes and triterpenes in the cell cytoplasm, is the most expressed among the prenyltransferases; its FPKM value is 337.79 (Fig. 5).

Diterpene biosynthesis typically consists of an initial cyclization of GGPP by a class II diTPS to produce a cyclic diphosphate intermediate, followed by conversion of this intermediate to the final diterpene skeleton by a class I diTPS [38]. *S. pomifera* unigene32268 that is abundantly expressed in trichomes (FPKM = 210.2), encodes for a protein that shares 97 % similarity (identities) to *S. fruticosa* copalyl diphosphate synthase (CDS) (GenBank: AJA38250.1), 90 % similarity to *R. officinalis* CDS (GenBank: AHL67261.1) and 87 % similarity to *S. miltiorrhiza* CDS (GenBank: AHJ59322.1). *S. pomifera* cl463 cluster of contigs code for a diterpene synthase that was previously characterized as miltiradiene synthase (GenBank: AJQ30185.1) [27]. CL463.contig4 that contains a full ORF identical to miltiradiene synthase is sufficiently expressed in trichomes of *S. pomifera* (FPKM =140.3).

### Identification, cloning and characterization of genes related to miltiradiene biosynthesis

**Fig. 6:**
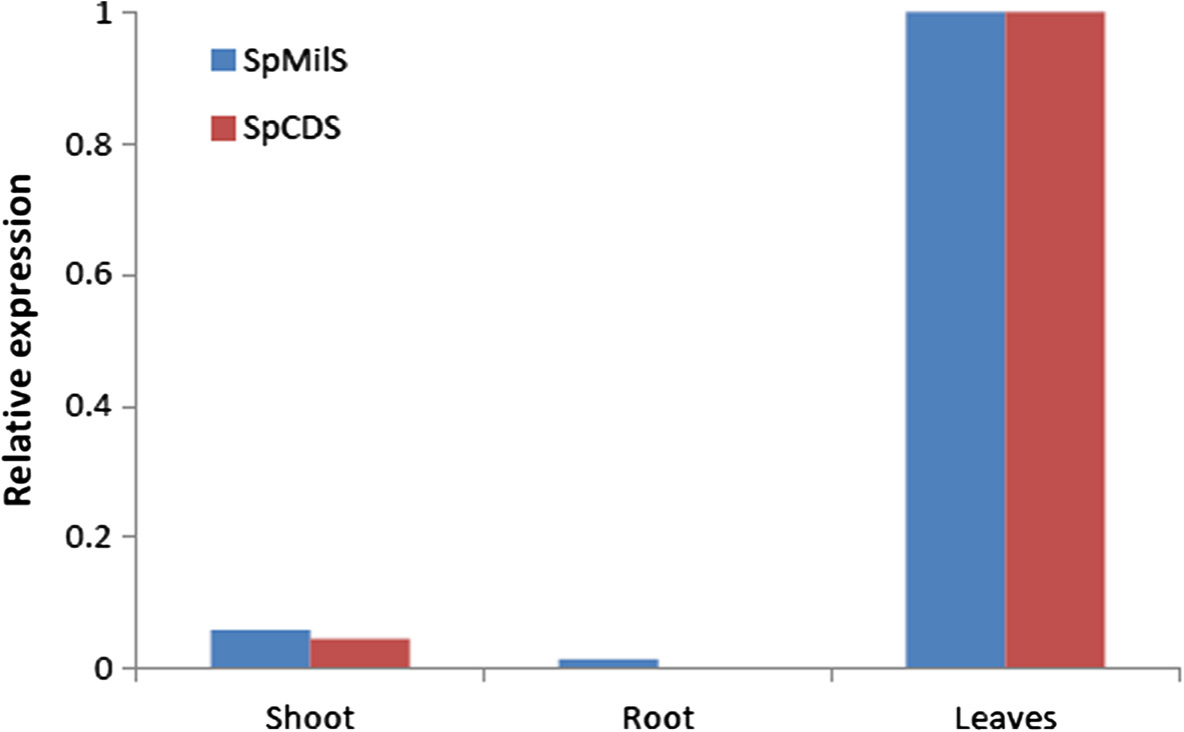
Relative expression of *S. pomifera* genes related to miltiradiene biosynthesis. Relative expression of miltiradiene synthase (SpMilS) and copalyl diphosphate synthase (SpCDS) in shoot, root and leaves of *S. pomifera*. Expression levels are depicted relatively in shoot and root compared to leaves expression

Expression analysis of unigene32268 and cl463 transcripts was performed by quantitative real time PCR (qPCR). Elongation factor 1-A was used as reference gene to normalize cDNA quantity. Expression analysis for the isolated genes was performed on leaves, root and shoot samples of *S. pomifera*. As a reference tissue sample leaves were selected. The expression levels of all the other tissues were expressed as relatively fold-differences to leaves. As shown in Fig. 6, unigene32268 is expressed only in leaves and more than 20 times less in the shoot of *S. pomifera*, while no expression was detected in the root. Cl463 expression levels are 100 times lower in the roots compared to leaves and more than 20 times lower in the shoot compared to the leaves (Fig. 6). High expression in leaves is anticipated; however the complete absence of expression of unigene32268 in roots is surprising considering that in extractions from root tissues we have identified sufficient amount of ferruginol (data not shown). It is likely that an alternative (tissue-specific) CDS gene may be responsible for labdane diterpene synthesis in the root.

**Fig. 7:**
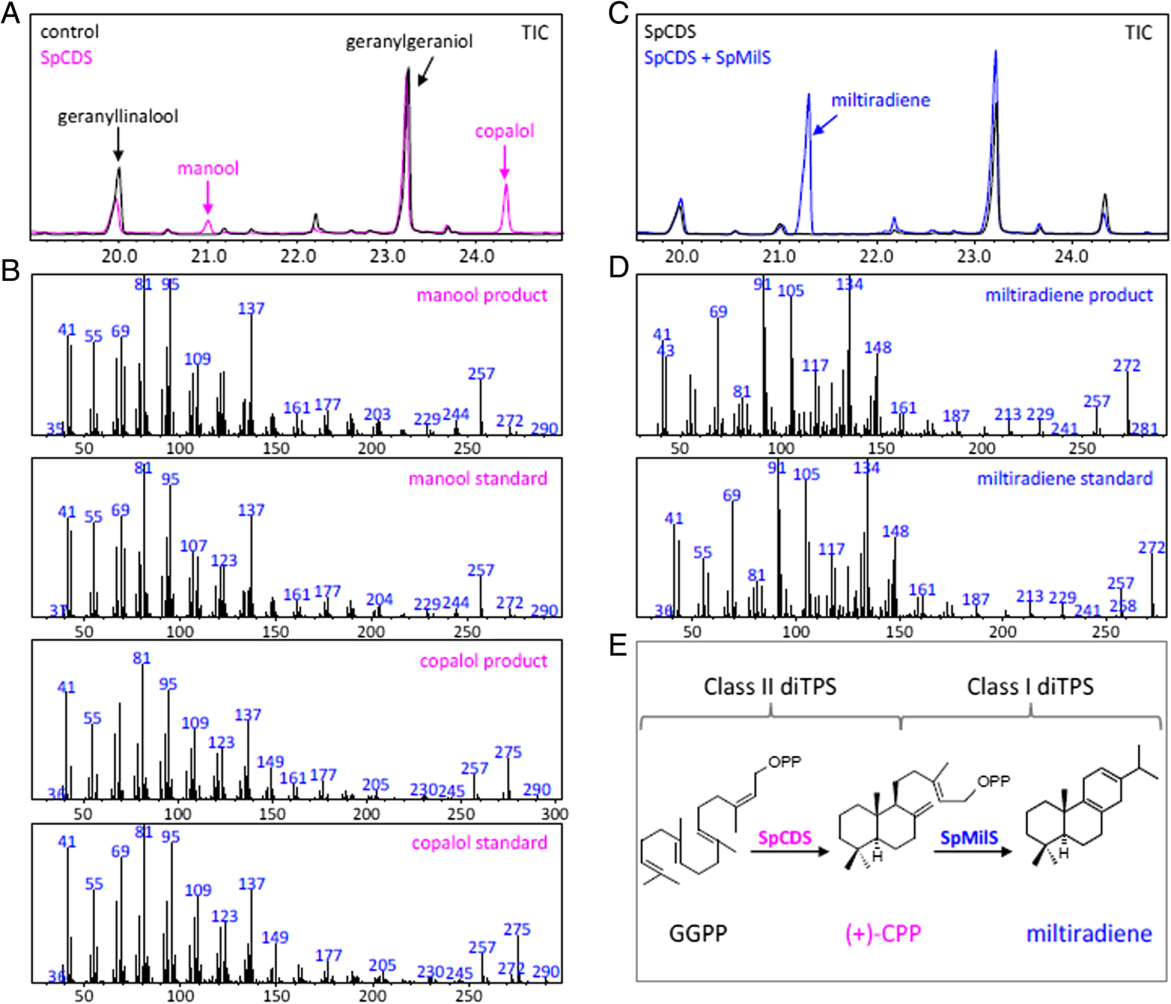
**a** Characterization of *S. pomifera* genes related to miltiradiene biosynthesis. *S. pomifera* Unigene 32268 encoding for a copalyl diphosphate synthase (SpCDS) expressed in AM238 yeast cell enabled production of manool and copalol as result of (+)-CPP hydrolysis. **b** Mass-spectrum of manool and copalol products and standards. **c** Production of miltiradiene in yeast cells co-expressing copalyl diphosphate synthase (SpCDS) and miltiradiene synthase (SpMilS). **d** Mass-spectrum of miltiradiene product and standard. **e** Biosynthetic pathway of miltiradiene in *S. pomifera* employ a class II diterpene synthase responsible for cyclization of the linear precursor geranylgeranyl diphosphate (GGPP) to form copalyl diphosphate ((+)-CPP), which is further converted to miltiradiene by a class I diterpene synthase

The *S. pomifera* unigene 32268 encoding for a putative copalyl diphosphate synthase (SpCDS) was PCR amplified using gene specific primers and introduced into the yeast expression vector pUTDH3myc, while the class I terpene synthase gene cl463 was amplified and cloned into pHTDH3myc [39]. For in vivo functional characterization, pseudomature protein was expressed in the yeast strain AM238 (MATα *his3, ura3, trp1, rox1, dos2, yer134c, vba5, ynr063w, ygr259c*) [40] together with the Erg20p (F96C) variant protein capable of improved production of GGPP [41]. Production of manool and copalol was observed in the yeast strain expressing the putative SpCDS. As proposed [41, 42], formation of manool and copalol is likely the result of acid or phosphatase-mediated hydrolysis, respectively, of excess of copalyl diphosphate (Fig. 7a). The identity of manool and copalol was verified by comparison of the retention time and mass-spectrum with authentic standard (Fig. 7b). Co-expression of the putative miltiradiene synthase (cl463) with SpCDS in AM238 yeast cells expressing the Erg20p(F96C) variant resulted in the production of miltiradiene (Fig. 7c), confirmed by comparison of the retention time and mass-spectrum with authentic standard (Fig. 7d). Miltiradiene titers under these conditions reached 32 mg/L (Fig. 7c), comparable with the previously reported ones [27, 43, 44]. Therefore, biosynthesis of miltiradiene was reproduced in heterologous yeast by employing the corresponding class II diterpene synthase, SpCDS, responsible for biosynthesis of copalyl diphosphate and the class I diterpene synthase, SpMilS (Fig. 7e).

### Cytochrome P450s

Cytochrome P450s is one of the largest families in plant metabolism and are responsible for the high chemical diversity of plant metabolites [45]. They are heme containing enzymes that catalyze the introduction of atmospheric oxygen into non-activated carbon-hydrogen bonds in a stereo- and regio-selective manner [46]. The transcriptomic analysis of *S. pomifera* trichomes revealed a number of CYPs either of full or partial length (Table 4). All CYPs were sent to Professor David Nelson for annotation [47]. The expression levels along with the annotation of *S. pomifera* CYPs are presented on Table 4. The highest transcribed CYPs are SpCYP76AH24 ortholog (GenBank: KT157044) (FPKM = 2026.30), SpCYP76AK6 (GenBank: KT157045) (FPKM = 1892.16), SpCYP71BE52 (GenBank: KT157042) (FPKM = 1746.15) and Sp716A96 (cv1) (GenBank: KT157037) (FPKM = 1326.01). Furthermore, several additional CYPs were identified having high expression levels such as SpCYP86A92 ortholog (GenBank: KT157046) (FPKM = 868.07), SpCYP71D455 (FPKM = 791.26), Sp73A120 ortholog (FPKM = 760.08) and SpCYP93B25 ortholog (FPKM = 721.56).

**Table 4:** Classification of *S. pomifera* CYPs according to their corresponding FPKM values produced by the RSEM software. The annotation of transcripts was kindly provided by Prof. David Nelson. All transcripts are full length unless otherwise stated; (cv) or (pv): complete or partial variant of the transcript

*S. pomifera* transcript	Annotation	Clan	FPKM
Unigene29490	CYP76AH24 ortholog	CYP71	2026.30
CL5059.Contig1	CYP76AK6	CYP71	1892.16
Unigene22114	CYP71BE52	CYP71	1746.15
CL528.Contig2	CYP716A96 (cv1)	CYP85	1326.01
Unigene69	CYP86A92 ortholog	CYP86	868.07
Unigene2012	CYP71D455	CYP71	791.26
Unigene5951	CYP73A120 ortholog	CYP71	760.08
Unigene32156	CYP93B25 ortholog	CYP71	721.56
CL2814.Contig2	CYP77A27-28 hybrid	CYP71	566.95
Unigene11611	CYP716A97 partial	CYP85	451.34
Unigene33285	CYP94A49 ortholog partial	CYP86	328.35
Unigene29149	CYP71D456 partial	CYP71	264.95
Unigene24154	CYP76G16 ortholog	CYP71	204.79
CL1793.Contig2	CYP79D48	CYP71	188.15
CL2247.Contig3	CYP96A107 partial	CYP86	182.21
CL2814.Contig1	CYP77A28 ortholog	CYP71	171.95
CL2247.Contig1	CYP96A85.1	CYP71	154.99
CL5680.Contig2	CYP98A75 ortholog	CYP71	150.89
CL648.Contig4	CYP76AH26 (pv1)	CYP72	124.85
Unigene27048	CYP82AL1	CYP71	115.16
CL9797.Contig1	CYP72A401	CYP71	92.27
CL4391.Contig1	CYP79D49	CYP74	74.64
Unigene31529	CYP71AU53 ortholog	CYP71	66.61
CL4021.Contig1	CYP74B21 ortholog (cv1)	CYP71	61.60
Unigene10824	CYP736A169	CYP72	58.17
CL8816.Contig2	CYP92B28 ortholog	CYP85	57.09
Unigene24932	CYP714G14	CYP71	56.05
CL3375.Contig3	CYP728D17 ortholog (cv1)	CYP71	55.01
Unigene28763	CYP75B79 ortholog	CYP85	54.06
CL528.Contig1	CYP716A96 (pv2)	CYP71	52.8
CL3177.Contig1	CYP71A64 hybrid (pv1)	CYP71	45.13
Unigene22595	CYP716C12 ortholog	CYP71	44.40
Unigene389	CYP84A61 ortholog	CYP86	41.33
Unigene26013	CYP89A115 ortholog	CYP72	40.45
CL67.Contig1	CYP84A60 ortholog	CYP71	34.61
CL3177.Contig2	CYP71A64 hybrid (pv2)	CYP86	31.75
CL9546.Contig1	CYP94C54 ortholog	CYP51	29.89
Unigene32931	CYP72A395 ortholog	CYP71	28.49
CL2274.Contig2	CYP81Q43 ortholog (cv1)	CYP71	28.22
CL8694.Contig1	CYP704A98 ortholog partial	CYP71	20.77
CL2247.Contig2	CYP96A85.2	CYP72	20.69
CL8331.Contig1	CYP51G1 ortholog partial	CYP97	20.68
Unigene30171	CYP71AT90 ortholog	CYP71	20.04
CL4021.Contig2	CYP74B21 ortholog (pv2)	CYP71	19.61
CL3621.Contig2	CYP76AH25 partial	CYP86	19.39
CL8143.Contig2	CYP76A35 ortholog (pv1)	CYP86	18.07
Unigene1656	CYP82U4 ortholog	CYP71	16.82
Unigene29994	CYP749A50 partial	CYP97	16.71
CL3665.Contig1	CYP97A41 ortholog partial	CYP72	15.83
CL913.Contig1	CYP71CS1 (GenBank: KT157039)	CYP71	15.75
CL2521.Contig1	CYP71A63	CYP71	14.63
CL2494.Contig1	CYP704A99 ortholog	CYP71	14.34
CL2454.Contig1	CYP94A48 ortholog partial	CYP72	11.90
CL3408.Contig2	CYP71AU68 (pv1) (GenBank: KT157040)	CYP72	10.53
CL3375.Contig2	CYP728D17 ortholog (pv2)	CYP72	9.83
CL9797.Contig2	CYP72A326 ortholog (pv1)	CYP71	9.69
CL4235.Contig2	CYP81B62 ortholog (pv1)	CYP85	9.68
Unigene29321	CYP97B34 ortholog	CYP71	9.04
CL2274.Contig1	CYP81Q43 ortholog (pv2)	CYP71	8.92
CL3449.Contig2	CYP714P1 (pv1)	CYP71	8.11
CL5917.Contig1	CYP81B77	CYP85	7.24
CL4235.Contig3	CYP81B62 ortholog (cv2)	CYP86	6.95
CL5645.Contig2	CYP76B64 (pv1)	CYP71	6.07
CL5645.Contig1	CYP76B64 (pv2)	CYP71	5.65
CL8094.Contig1	CYP72A400	CYP71	4.76
CL3449.Contig1	CYP714P1 (pv2)	CYP71	4.35
CL3408.Contig1	CYP71AU68 (pv2)	CYP85	3.26
CL2016.Contig4	CYP72A393 ortholog (pv1)	CYP86	3.23
CL3361.Contig1	CYP749A49 partial	CYP71	3.17
CL648.Contig3	CYP76AH26 (pv2)	CYP71	2.99
CL2016.Contig3	CYP72A393 ortholog (pv2)	CYP71	1.85
CL8143.Contig1	CYP76A35 ortholog (cv2) (GenBank: KT157043)	CYP86	1.71
CL9797.Contig3	CYP72A326 ortholog (pv2)	CYP71	1.51
CL908.Contig1	CYP707A102 ortholog partial	CYP71	0.79
CL3449.Contig3	CYP714P1 (pv3)	CYP72	0.02
CL648.Contig1	CYP76AH26 (pv3)	CYP71	0.00
CL648.Contig2	CYP76AH26 (pv4)	CYP71	0.00
Unigene34025	CYP76AH26 (pv5)	CYP71	0.00
Unigene34026	CYP76AH26 (pv6)	CYP71	0.00
Unigene34027	CYP76AH26 (pv7)	CYP71	0.00
Unigene34028	CYP76AH26 (pv8)	CYP71	0.00

The highest transcribed CYP, SpCYP76AH24 ortholog (FPKM = 2026.30), was found to be homologous (identity 80 %) to *S. miltiorrhiza* CYP76AH1 (GenBank: AGN04215.1) that catalyzes the synthesis of ferruginol [22]. SpCYP76AH24 ortholog encodes a predicted protein of 492 amino acids that is almost identical (identity 99 %) to the ferruginol synthase from *S. fruticosa* (GenBank: AJQ30186.1) [27] and highly homologous (identity 91 %) to *CYP76AH4,* the ferruginol synthase from *R. officinalis* (GenBank: AJQ30187.1) [23]. It is suggested that miltiradiene spontaneously aromatizes to abietatriene, which is then hydroxylated by ferruginol synthase to yield ferruginol, the precursor of other labdane-related terpenes like carnosol and tanshinones [23].

**Fig. 8:**
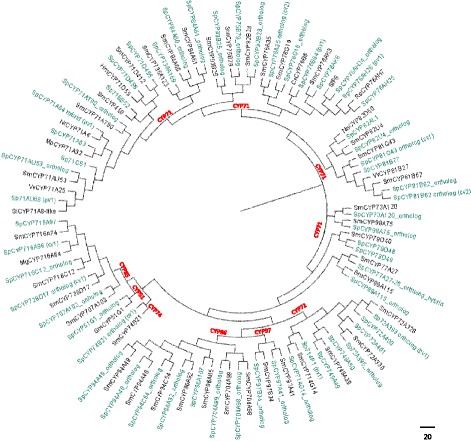
A neighbor-joining tree of *S. pomifera’s* CYPs. Phylogenetic analysis of *S. pomifera* cytochrome P450s. Green labels represents *S. pomifera* CYPs and red labels represent the different clans. Among gene variants the full length one (cv) was chosen when possible otherwise the most transcipted partial variant (pv)

A phylogenetic analysis of CYPs revealed that the majority belong to the CYP71 clan while the rest of transcripts are categorized to the clans CYP72, CYP85 and CYP86. Clans CYP51 and CYP74 have one member each while CYP97 has two members as demonstrated on the phylogenetic tree (Fig. 8). The clustering of families of *S. pomifera’s* CYPs on the phylogenetic tree comes to verify the rooted relationships of clans. For instance, the proximity of CYP97 with CYP86 and CYP51 with CYP85 implies a possible evolution from a common ancestor [45]. Members of the CYP71 clan were shown to be close phylogenetically with enzymes involved in isoprenoid biosynthesis. Two members of the CYP76 family, Sp76AK6 and Sp76G16 ortholog are phylogenetically close with Sm76AK3 and Sm76G16, respectively, suggesting an involvement in diterpenoid biosynthesis [48]. On the other hand, SpCYP71D456 showed high similarity with CYP71D55 from *Hyoscyamus muticus* that is designated as premnaspirodiene oxidase [49] in sesquiterpenoid biosynthesis. Regarding monoterpenes, SpCYP71AU53 ortholog (GenBank: KT157041) and SpCYP81B62 ortholog are probably involved in the biosynthesis of monoterpenoids [48]. Along with clan CYP71, one member from CYP85 clan, SpCYP728D17 ortholog (GenBank: KT157038) is probably involved in diterpenoid biosynthesis [48]. From the CYP85 clan, according to the computational expression analysis, the highest expressed transcripts are Sp716A96 (1) (FPKM = 1326.01) and SpCYP716A97 (FPKM = 451.34); both belonging to CYP716A subfamily which have been shown to be involved in diterpenoid [48] and triterpenoids biosynthesis [50]. In general, members of the CYP85 clan have been shown to be involved in the metabolism of medium to large isoprenoids [45]. The majority of clan CYP86 members are orthologous to their counterparts from *S. miltiorrhiza* [48] with SpCY96A92 ortholog, SpCYP94A49 ortholog and SpCYP96A107 having the highest expression among them.

Besides isoprenoid metabolism, specific transcripts were identified to be orthologous to *S. miltiorrhiza’s* CYPs that are probably involved in rosmarinic acid pathway. Sp73A120 ortholog and SpCYP98A75 ortholog have been identified as cinnamate 4-hydroxylase and β-coumaryl-CoA 3′-hydroxylase respectively, two key enzymes in rosmarinic acid biosynthesis in *S. miltiorrhiza* [48, 51].

## Discussion

Labdane diterpenes have important health promoting properties. Carnosic acid-related compounds from rosemary and sage have attracted attention not only as potent antioxidants but also as antibacterial, antiparasitic and cytotoxic compounds [33, 52–56] while tanshinones from *S. miltiorrhiza* roots have a plethora of bioactive properties [7, 9, 10, 12, 57]. The biosynthesis of carnosic acid and tanshinones is still unclear. Although ferruginol appears to be a common intermediate, the following steps and the point where these pathways disentangle are not yet known. Interestingly, it has been proposed that tanshinone biosynthesis may proceed through carnosic acid [22], although this claim has not since been validated. Elucidating the biosynthesis of these compounds will be an important step towards their biotechnological production in engineered microorganisms.

In the present study, thorough metabolite analysis of *S. pomifera* leaves led to the isolation and identification of several metabolites from the carnosic acid family (Fig. 1). To elucidate their biosynthesis, the transcriptome of *S. pomifera* glandular trichomes was sequenced to obtain approximately 54,000,000 raw reads that were assembled into contigs and annotated. Expression analysis of the transcripts involved in the isoprenoid biosynthesis showed higher expression of the genes participating in the MVA pathway in comparison to the MEP pathway. These findings are in contrast to previous observations from *S. miltiorrhiza* where it was shown that MEP related transcripts were mainly expressed in the leaf while MVA-related transcripts were mainly expressed in the root [24].

In addition to isoprenoid biosynthesis, several highly expressed transcripts that were identified in the trichomes of *S. pomifera* are highly similar to *S. fruticosa* ones indicating that they probably play important roles in this specialized tissue which could be investigated further. Other contigs that code for photosynthetic related proteins such as ribulose biphosphate carboxylase (Rubisco) and chlorophyll-binding proteins are among the most expressed in trichomes. Similarly, such ESTs coding for proteins involved in photosynthesis have also been found among the most expressed genes in the trichomes of *Artemisia annua*, the Asian medicinal plant that produces the sesquiterpene lactone artemisinin [58]. The abundant expression of photosynthesis related genes in the trichomes transcriptome of *S. pomifera* is also in accordance with data from other plant species such as several *Solanum* species - both wild and domesticated - in which photosynthetic genes were also highly expressed in glandular trichomes [59]. *S. pomifera* trichomes are possibly capable of synthesizing secondary metabolites without the need for carbon transport from leaves. The opposite has been suggested by Jin et al. [37] for the peltate glandular trichomes of spearmint, *M. spicata*, described as poor in genes related to photosynthesis and chlorophyll formation. We proceeded to reassemble and analyze the deposited *M. spicata* transcriptome data and compared them to the *S. pomifera* ones. Data analysis of the *M. spicata* transcriptome sequencing showed that two genes coding for Rubisco and chlorophyll binding proteins are also highly expressed in the trichomes of *M. spicata*. The common high expressed genes between *S. pomifera* and *M. spicata* are indicated in bold in the Additional file 9: Table S1 that includes the complete list of *M. spicata* most expressed transcripts as a result of our analysis. With regard to photosynthetic competence, the possibility of leaf cells contaminating trichome samples cannot be ruled out, though no such tissue could be seen by visual and microscopic observation of the trichomes destined for *S. pomifera* NGS sequencing.

The transcriptomic analysis pointed two highly expressed diterpene synthases, SpCDS and SpMilS, that were cloned and expressed in the appropriate yeast platform yielding 32 mg/L miltiradiene, that is in accordance with previously reported ones [27, 43, 44] (Fig. 7c). The abundance of SpCDS and SpMilS was further supported from the expression analysis by qPCR in three different tissue organs of *S. pomifera* (Fig. 6) that showed high expression in leaves and poor or zero expression in other tissues. This might be explained by the fact that carnosic acid is mainly stored in the glandular trichomes of the leaves [26, 27], thus a higher expression of the relevant enzymes is expected. On the contrary, their homologues in *S. miltiorrhiza*, SmCPS and SmKSL, were shown to be abundantly expressed in roots, which is expected since tanshinones accumulation takes place in that specific tissue [24].

Along with terpene synthases, CYPs are the key enzymes contributing to the diversification of terpenoids. They are a highly diverse family of enzymes catalyzing various reactions [60] responsible for the chemical diversity of secondary metabolites of the plant kingdom. Terpene synthases create the terpene scaffold giving rise to a variety of metabolites [61], while CYPs undertake further modification of the scaffold increasing the diversity of the resulting terpenes. Study of terpene synthases along with CYPs is essential since a non random functional association of these two families has been revealed [62]. Thus, having available the whole transcriptomic profile of CYPs is of a great significance.

The transcriptomic analysis of *S. pomifera* trichomes revealed a significant number of highly transcribed CYPs, with SpCYP76AH24 ortholog being the highest among them. SpCYP76AH24 ortholog showed high similarity to ferruginol synthases from other Lamiaceae species; 99 % identity to *S. fruticosa* ferruginol synthase, 91 % identity to *R. officinalis* CYP76AH4 and almost 80 % homology to *S. miltiorhhiza* CYP76AH1. The predicted protein from SpCYP76AH24 ortholog has a full pP450 domain (Pfam family: PF00067) that extends from position 28 to position 463 and due to its similarity with the known Lamiaceae CYP76AHs, it is highly possible to produce ferruginol in *S. pomifera.*

Phylogenetic analysis of cytochrome P450s showed that the majority of CYPs belong to CYP71, CYP72, CYP85 and CYP86 clans as was anticipated [50]. It was previously shown that CYPs involved in the isoprenoid biosynthesis likely originate from CYP71 and CYP85 clan. CYP71 clan members are mainly involved in the metabolism of specialized metabolites like monoterpenes and sesquiterpenes substrates [50] as well as in the metabolism of aromatic and aliphatic amino acids, alkaloids, fatty acids and precursors of hormones [45]. CYP71 clan includes the majority of the CYP450 families that are involved in the plant secondary metabolism [50, 60]. CYP71A family proteins participate in monoterpenoids oxidation even though it has been suggested that may be highly specialized in their roles in diverse plant species [50]. On the other hand, CYP76 family is mainly involved in isoprenoid metabolism [60] although members of this family have also been implicated in herbicide metabolism in *Arabidopsis* [63] and in the biosynthesis of defense compounds in *Thuja plicata* [64]. Clans CYP85 and CYP86 are involved in the metabolism of both general and specialized metabolites [50]. Besides isoprenoid metabolism, two member enzymes were annotated as orthologous to SmCYP98A75 and SmCYP73A120 that are - a β-coumaryl-CoA 3′-hydroxylase and a cinnamate 4-hydroxylase respectively, key enzymes in rosmarinic acid biosynthesis [48, 51].

## Conclusions

Over the past years, tanshinones and carnosic family metabolites, have gained great attention due to their unique properties. Thus, elucidation of their biosynthetic pathway is of great importance as it will unlock the key enzymes involved in their synthesis. The transcriptome analysis of *S. pomifera* trichomes revealed two genes involved in miltiradiene synthesis; a copalyl diphosphate synthase (SpCDS) and a miltiradiene synthase (SpMilS). Furthermore, the phylogenetic analysis of several cytochrome P450s provided useful insights in the biosynthetic mechanism responsible for the production of carnosic acid-family metabolites in *S. pomifera.* Setting up a resource of CYP450 genes from *S. pomifera* not only provides information on terpenoid biosynthesis but also assists in mining genes involved in the production of other natural products.

## Methods

### Chemicals

n-Hexane (≥99 %) and diethyl ether (≥99.8 %) used for plant extract and GC/MS analysis respectively were both purchased from Sigma-Aldrich (St. Louis, MO, USA). Cyclohexane, CH_2_Cl_2_, acetone and acetic acid ethyl ester (EtOAc) were all purchased from Sigma-Aldrich (St. Louis, MO, USA).

MyTaq DNA polymerase (BIO-21105, Bioline, Taunton, USA), and Accuzyme DNA polymerase (BIO-21051, Bioline, Taunton, USA) were used in PCR amplifications; NucleoSpin Plasmid Kit (REF 740588.250, Macherey-Nagel, Düren, Germany) was used for plasmid DNA purification; QIAquick Gel Extraction Kit (#28704, Qiagen, Hilden, Germany) was used for gel extraction and DNA purification.

Yeast media: D (+)-Glucose monohydrate (Sigma-Aldrich, St. Louis, MO, USA); Yeast Nitrogen Base w/o AA, carbohydrate & w/AS (Y2025, US Biologicals, Life Sciences, Texas, USA); Complete Minimal (CM) medium is composed of 0.13 % (w/v) dropout powder (all essential amino acids), 0.67 % (w/v) yeast nitrogen base w/o AA, 2 % glucose; TOPO TA Cloning Kit Dual Promoter (K4610-20, Life Technologies, Carlsbad, CA, USA). All yeast transformations were done by lithium acetate transformation.

### Plant material

*S. pomifera* plants were grown from seeds collected from the wild populations of Crete, Greece (Topolia and Aptera area). Plants were grown in the research greenhouse in the farm of Aristotle University of Thessaloniki, Greece, under controlled temperature (25/18 °C, day/night, winter, 32/20 °C, day/night, summer) and natural photoperiod.

### Plant extraction and isolation of metabolites

The aerial parts of *S. pomifera* plants grown from seeds collected from the areas of Topolia and Aptera, Grete, Greece were separately extracted with EtOAc at room temperature. Evaporation of the solvent under vacuum afforded green brownish organic residue.

The organic residue of Aptera (SP1, 607 mg) was subjected to vacuum column chromatography on Sephadex LH-20, using cyclohexane/CH_2_Cl_2_ (1:4 v/v), CH_2_Cl_2_/acetone (3:2 v/v) and CH_2_Cl_2_/acetone (1:4 v/v) as mobile phase, to yield 11 fractions (SP1f1–SP1f11). Fraction SP1f7 (cyclohexane/CH_2_Cl_2_ 1:4 v/v, 16.3 mg) was further fractionated by gravity column chromatography on silica gel, using cyclohexane with increasing amounts of EtOAc as the mobile phase, to afford 4 fractions (SP1f7a–SP1f7d). Fraction SP1f7c (15 % v/v EtOAc in cyclohexane, 5.2 mg) was purified by normal phase HPLC, using cyclohexane/acetone (85:15 v/v) as eluent, to yield metabolite **2** (1.3 mg). Fraction SP1f8 (CH_2_Cl_2_/acetone 3:2 v/v, 64.9 mg) was further fractionated by gravity column chromatography on silica gel, using cyclohexane with increasing amounts of EtOAc as the mobile phase, to afford 5 fractions (SP1f8a–SP1f8e). Fraction SP1f8b (15 % v/v EtOAc in cyclohexane, 39.6 mg) was purified by normal phase HPLC, using cyclohexane/acetone (85:15 v/v) as eluent, to yield metabolites **4** (12.2 mg) and **5** (1.6 mg). Fraction SP1f9 (CH_2_Cl_2_/acetone 3:2 v/v, 57.6 mg) was further fractionated by gravity column chromatography on silica gel, using cyclohexane with increasing amounts of EtOAc as the mobile phase, to afford 6 fractions (SP1f9a–SP1f9f). Fraction SP1f9d (25 % v/v EtOAc in cyclohexane, 27.9 mg) was purified by normal phase HPLC, using cyclohexane/acetone (83:17 v/v) as eluent, to yield metabolites **1** (17.0 mg) and **6** (10.0 mg).

The organic residue of Topolia (SP4, 223 mg) was subjected to vacuum column chromatography silica gel, using cyclohexane with increasing amounts of EtOAc as mobile phase, to yield 10 fractions (SP4a–SP4b). Fraction SP4f (12 % v/v in EtOAc, 50 mg) was subjected to normal phase HPLC, using cyclohexane with increasing amounts of EtOAc as mobile phase, to yield 9 fractions (SP4f1–SP4f9). Fraction SP4f5 (26.0 mg) was further fractionated by gravity column chromatography on silica gel, using cyclohexane with increasing amounts of EtOAc as the mobile phase, to afford 5 fractions (SP4f5a–SP4f5e). Fraction SP4f5b (10 % v/v EtOAc in cyclohexane, 18.5 mg) was purified by normal phase HPLC, using cyclohexane/EtOAc (86:14 v/v) as eluent, to yield metabolite **3** (1.6 mg). Fractions SP4f6 to SP4f9 (12, 15, 20 and 30 % v/v EtOAc in cyclohexane, respectively) were separately subjected to normal phase HPLC using a gradient mobile phase as described below. The peaks corresponding to the elution times of the isolated compounds were collected and were further purified by normal phase HPLC using the same conditions as described for SP1 to afford metabolites **1** (0.6 mg), **2** (4.1 mg), **4** (0.7 mg) and **6** (2.3 mg). The isolated compounds (**1**–**6**) were subjected to normal phase HPLC analysis using a gradient elution system. Their elution times were determined and their characteristic UV spectra were also recorded, both serving as reference data for the identification of the compounds within the extracts. The mobile phase of the gradient system consisted of cyclohexane (A) and EtOAc (B). A flow rate of 1.5 mL/min was applied, using the following gradient profile: 10 % B up to 100 % B within 50 min and then the solvent was kept constant to 100 % B for 10 min. All organic residues were qualitatively analyzed according to the described method.

#### 2*α*-hydroxy-*O*-methyl-pisiferic acid (1)

White solid; [*α*] $$ \begin{array}{c}\hfill 20\hfill \\ {}\hfill \mathrm{D}\hfill \end{array} $$ +93.0 (*c* 0.17, CHCl_3_); UV (CHCl_3_) *λ*_max_ (log *ε*) 243 (2.62), 284 (2.41) nm; IR (film) *ν*_max_ 3316, 2966, 22918, 2852, 1679, 1508, 1459, 1247, 1027; NMR data, see Table 1; EIMS (70 eV) *m/z* (rel. int. %) 346 (76), 301 (13), 283 (100), 241 (12), 159 (7); HR-ESIMS *m/z* 345.2066 [Μ-H]^−^ (calcd for C_21_H_29_O_4_, 345.2071).

#### General experimental procedures

Optical rotations were measured on a Perkin-Elmer model 341 polarimeter with a 1 dm cell. UV spectra were recorded on a Perkin Elmer Lambda 40 spectrophotometer. IR spectra were obtained on a Bruker Tensor 27 spectrometer. NMR spectra were recorded on Bruker AC 200 and Bruker DRX 400 spectrometers. Chemical shifts are given on a *δ* (ppm) scale using TMS as internal standard. The 2D experiments (HSQC, HMBC, COSY, NOESY) were performed using standard Bruker pulse sequences. High resolution ESI mass spectra were measured on a Thermo Scientific LTQ Orbitrap Velos mass spectrometer. Column chromatography separations were performed with Kieselgel 60 (Merck, Kenilworth, USA) and Sephadex LH-20 (Pharmacia & Upjohn Company LLC, USA). HPLC separations were conducted using a Pharmacia LKB 2248 liquid chromatography pump equipped with a RI-102 Shodex refractive index detector, using a Supelcosil SPLC-Si 5 μm (250 × 10 mm i.d.; Supelco) column. HPLC gradient elution separations for qualitative analysis were conducted using a Waters 600 Controller pump equipped with a Waters 996 Photodiode Array detector, using Kromasil Silica 8 μm (250 × 10 mm i.d.; AkzoNobel) column. TLC was performed with Kieselgel 60 F_254_ (Merck aluminum-backed plates) and spots were detected after spraying with 15 % H_2_SO_4_ in MeOH reagent and heating at 100 °C for 1 min.

### RNA extraction from *S. pomifera* leaf trichomes

Leaf material from *S. pomifera* Topolia was frozen in liquid nitrogen immediately after collection and was kept in −80 °C until processing. Leaf trichomes were isolated with the dry ice abrasion method [65]. High quality total RNA from the plant trichomes was isolated using the commercial Spectrum Plant Total RNA Kit (Sigma Aldrich, St. Louis, MO, USA), according to the manufacturers’ specifications. Quality control of RNA was performed using an Agilent 2100 Bioanalyzer.

For Illumina sequencing, at least 20 μg of RNA was used satisfying the following requirements: concentration ≥ 400 ng/μl, OD260/280 = 1.8 ~ 2.2; OD260/230 ≥ 2.0; RNA 28S:18S > 1.0 and RIN ≥ 7.0.

### Transcriptome sequencing, *de novo* assembly and gene annotation

cDNA synthesis and transcriptome sequencing was performed by the Beijing Genomic Institute, Shenzen, China, using an Illumina Hiseq™ 2000 platform.

The obtained raw reads were cleaned from low quality reads, reads that contained adaptors and unknown nucleotides larger than 5 % using fastqc and cutadapt coordinated by the wrapper Trim_Galore (Version 0.3.7). *De novo* assembly of high quality reads into unigenes was carried out using the Trinity suite [34] using the default parameters for paired end sequenced libraries.. The resulted output from Trinity contained two classes of contigs; the first class, contained clusters of unigenes with similarity higher than 70 % (prefix cl) and the second class contained singletons (prefix unigene).

In order to decide sequence direction of unigenes, a BlastX alignment (e value < 10^−5^) between unigenes and protein databases like nr (non redundant nucleotide database, NCBI), Swiss-Prot, KEGG (Kyoto Encyclopedia of Genes and Genomes) and COG (Clusters of Orthologous Groups) was performed and the best alignment result was chosen. If there was a confliction between results, the following order of databases was followed for the sequence direction of unigenes: nr, Swiss-Prot, KEGG and COG. When a unigene was not aligned to any of the above databases, the software ESTScan was employed [66].

Functional annotation of unigenes was performed using nr, Swiss-Prot, KEGG and COG databases. For the GO annotation of unigenes based on nr database, the annotation software Blast2GO [67] was used while the GO functional classification of all unigenes was performed with WEGO software [68]. Transcript quantification was estimated from RNA-Seq data using the RSEM software package contained in the Trinity suite. Unigenes were used as reference to estimate the abundance of expression based on the paired-end RNA-Seq data using the standard instructions and parameters as described in http://deweylab.biostat.wisc.edu/rsem/README.html and in http://trinityrnaseq.sourceforge.net/analysis/abundance_estimation.html.

### Phylogenetic analysis

The evolutionary history was inferred using the Neighbor-Joining method [69]. The bootstrap consensus tree inferred from 1000 replicates [70] is taken to represent the evolutionary history of the taxa analyzed [70]. Branches corresponding to partitions reproduced in less than 50 % bootstrap replicates are collapsed. The evolutionary distances were computed using the p-distance method [71] and are in the units of the number of amino acid differences per site. The analysis involved 109 amino acid sequences of CYPs. All ambiguous positions were removed for each sequence pair. There were a total of 788 positions in the final dataset. Evolutionary analyses were conducted in MEGA6 [72].

### Yeast strain, expression vectors and miltiradiene analysis

The *S. pomifera* unigene 32268 encoding for a putative copalyl diphosphate synthase (SpCDS) was PCR amplified using gene specific primers and introduced into the yeast expression vector pUTDH3myc [39]. For in vivo functional characterization, pseudomature SpCDS protein was expressed in the yeast strain AM238 [Mat α *his3, ura3, trp1, rox1, dos2, yer134c, vba5, ynr063w, ygr259c*] [40] also expressing the Erg20p(F96C) variant protein capable of improved production of GGPP [41]. The SpMils construct into the yeast expression vector has been described previously [44].

For the determination of SpCDS and SpMilS products, 5 ml cultures of the relevant yeast strain were grown overnight at 30 °C in the appropriate selection media rotating in a roller drum. The next day 500 μl of decane (1:10 v/v) was added to each tube, and the cultures were incubated for two additional days. At the end of the incubation period the organic layer was transferred into eppendorf tubes, centrifuged to separate any residual medium left, and 200 μl were loaded on GC vials and analyzed by GC-MS for product identification and GC-FID for quantification.

### Expression analysis in different tissues of *S. pomifera*

Total RNA was extracted from leaves, root and shoot samples of *S. pomifera* using the commercially available kit Spectrum™ Plant Total RNA Kit (Sigma-Aldrich, St. Louis, MO, USA) according to the manufacturer’s instructions. Total RNA from all tissues were subjected to DNase I treatment (Life Technologies, Carlsbad, CA, USA) in order to remove any traces of simultaneously isolated DNA. One μg of total RNA from all tissues were used in a reverse transcription reaction using random hexamers and SuperScript II-RT (Life Technologies, Carlsbad, CA, USA) following the manufacturers protocol. For every tissue an identical NO-RT reaction was included in which Supercript II reverse transcriptase was replaced with DEPC-treated water. Real-time quantitative PCR was performed on the Rotorgene-Q 5plex Platform (using the Kapa SYBR® Fast qPCR Kit Master Mix (2X) Universal KapaBiosystems, Capetown, South Africa) with 1/20 of the synthesized cDNAs as template and the corresponding primers at 0.4 μM. The cycling conditions were: 3 min 95 °C, 35 cycles of 5 s/95 °C, 30 s/64 °C and a melting step of 65–95 °C with a ramp rate of temperature increase of 0.1 °C with a hold of 2 s. The primers for miltiradiene synthase (MilS) were P1326-F: 5′-GGCGCAGTGTCAAAGACTTTCTAA-3′ and P1326-R: 5′-CCATCGAAACATGCCTACACAAAT-3′, for copalyl diphosphatase synthase (CDS) SpCDS-F: 5′- CCGAGTTGAGGCTGCCTATTATCT-3′ and SpCDS-R: 5′-CGAATTCACTAACGCTGCTTCTGT-3′ were designed using the Primer 3.0 online tool (http://biotools.umassmed.edu/bioapps/primer3_www.cgi). A fragment of the Elongation Factor 1a gene was also amplified in order to validate successful cDNA synthesis and for normalization purposes. The primers used were designated SpEF1a-F: 5′- GTTGCCTCTAACTCCAAGGACGAT-3′ and SpEF1a-R: 5′- CCAGCATCACCATTCTTCAAAAAC-3′. All reactions were performed in triplicates. Relative quantification was calculated using the 2^-ΔΔCt^ algorithm a convenient method to analyze the relative changes in gene expression which requires the assignment of one or more housekeeping genes (assumed to be uniformly and constantly expressed in all samples, as well as one or more reference samples). The expression of other samples is then compared to that in the reference sample [73].

### Availability of data

Illumina Hiseq 2000 raw transcriptome sequences and Transcriptome Shotgun Assembly are available at NCBI Bioproject database under the accession number PRJNA292070 and the SRA database under the Run Experiment accession number SRR2136651.

## Additional files

Additional file 1: Figure S2.
^1^H-NMR spectrum of 2*α*-hydroxy-*O*-methyl-pisiferic acid (compound 1). (PDF 114 kb) Additional file 2: Figure S3.
^13^C-NMR spectrum of 2*α*-hydroxy-*O*-methyl-pisiferic acid (compound 1). (PDF 271 kb) Additional file 3: Figure S4.
^1^H-NMR spectrum of pisiferic acid (compound 2). (PDF 116 kb) Additional file 4: Figure S5.
^1^H-NMR spectrum of *O*-methyl-pisiferic acid (compound 3). (PDF 144 kb) Additional file 5: Figure S6.
^1^H-NMR spectrum of 12-methoxycarnosic acid (compound 4). (PDF 111 kb) Additional file 6: Figure S7.
^1^H-NMR spectrum of carnosol (compound 5). (PDF 116 kb) Additional file 7: Figure S8.
^1^H-NMR spectrum of salviol (compound 6). (PDF 113 kb) Additional file 8: Figure S1. (A) Distribution of *S. pomifera* contigs according to their length and (Β) Distribution of *S. pomifera* clusters of contigs and unigenes according to their length. (PDF 111 kb) Additional file 9: Table S1. Thirty-five most expressed genes/contigs in *M. spicata* trichomes based on their FPKM values produced by the RSEM software. In the last column their top hit is referred, produced by BLAST searches in the NR database. The length of the contigs is sometimes not an integer number due to the fact that one contig may correspond to several transcripts of diverse length. The RSEM software was run on the data produced by the transcriptome sequencing of *M. spicata* trichomes by Jin et al. [37]. (PDF 103 kb)
