# Supplementary figures and images for: Combined metabolome and transcriptome profiling provides new insights into diterpene biosynthesis in S. pomifera glandular trichomes
Source: BMC Genomics. 2015 Nov 14;16:935. doi: 10.1186/s12864-015-2147-3 (PMC4647624; doi:10.1186/s12864-015-2147-3)

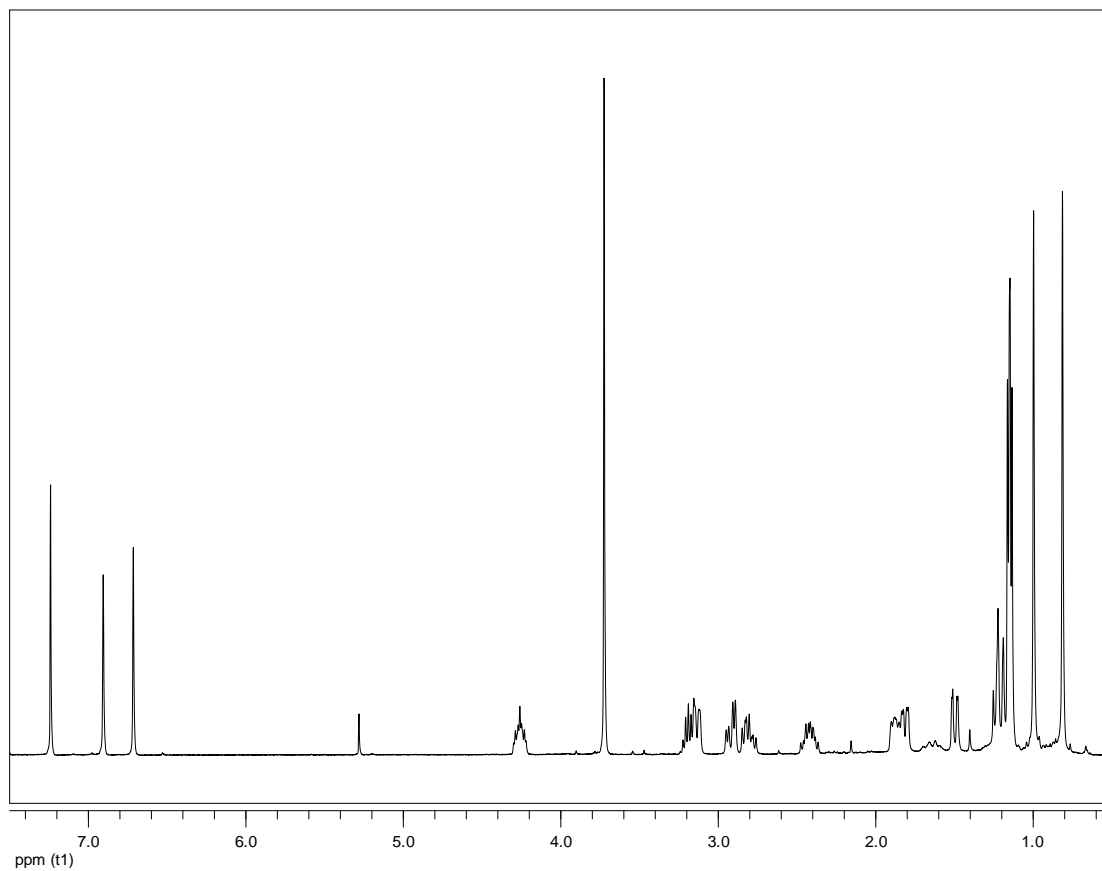

Supplement: Additional file 1: Figure S2. — 1H-NMR spectrum of 2α-hydroxy-O-methyl-pisiferic acid (compound 1). (PDF 114 kb) [file 12864_2015_2147_MOESM1_ESM.pdf]

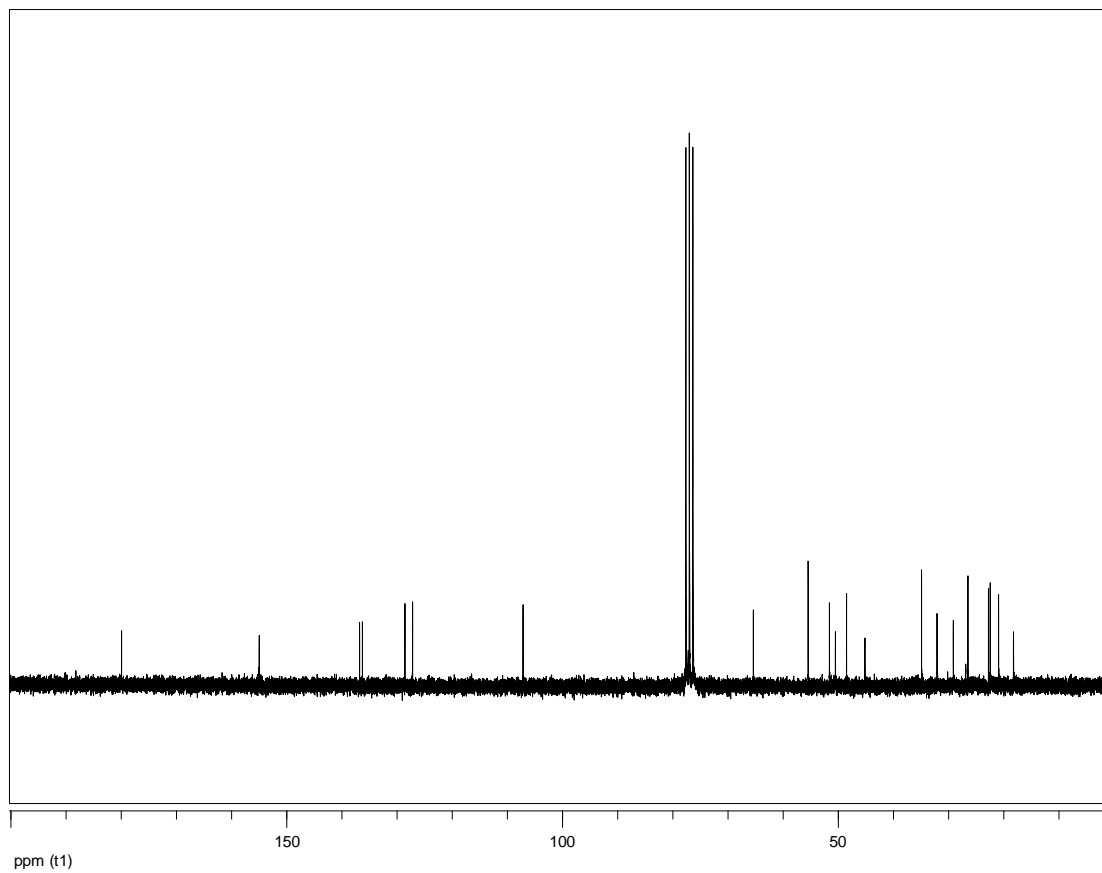

Supplement: Additional file 2: Figure S3. — 13C-NMR spectrum of 2α-hydroxy-O-methyl-pisiferic acid (compound 1). (PDF 271 kb) [file 12864_2015_2147_MOESM2_ESM.pdf]

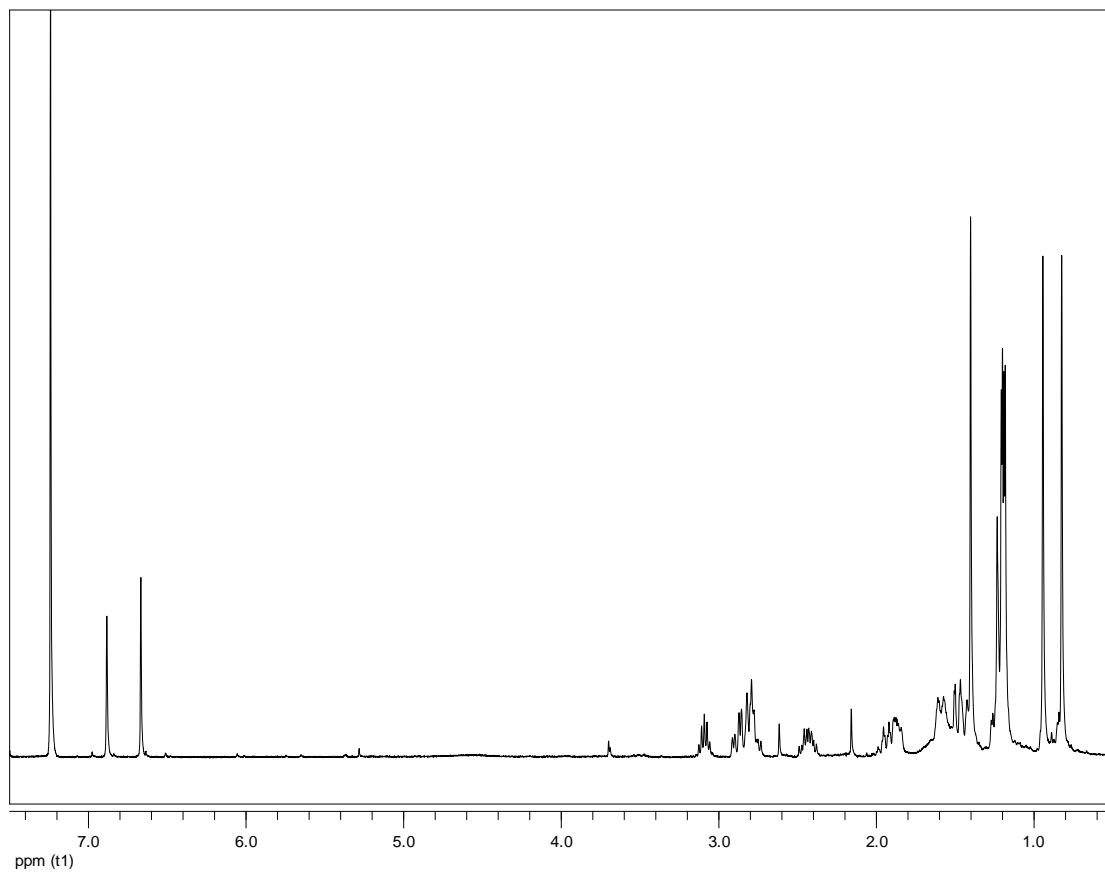

Supplement: Additional file 3: Figure S4. — 1H-NMR spectrum of pisiferic acid (compound 2). (PDF 116 kb) [file 12864_2015_2147_MOESM3_ESM.pdf]

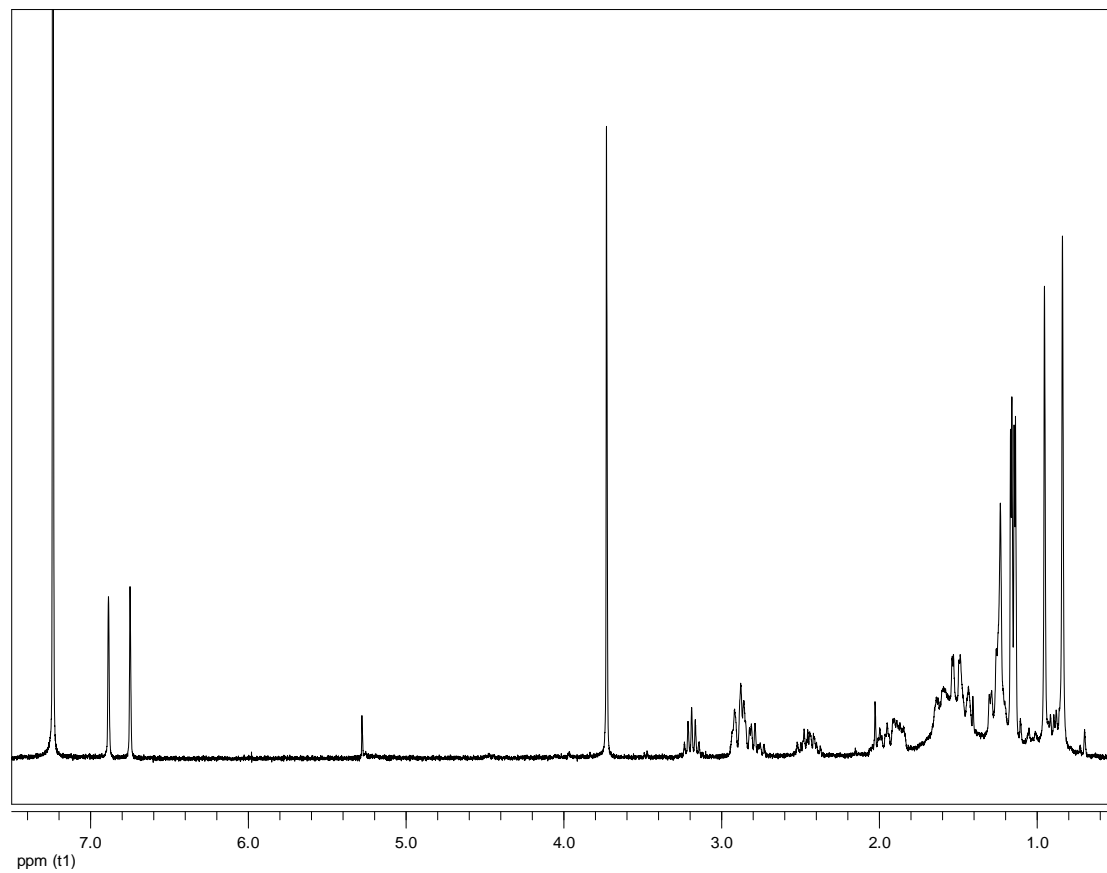

Supplement: Additional file 4: Figure S5. — 1H-NMR spectrum of O-methyl-pisiferic acid (compound 3). (PDF 144 kb) [file 12864_2015_2147_MOESM4_ESM.pdf]

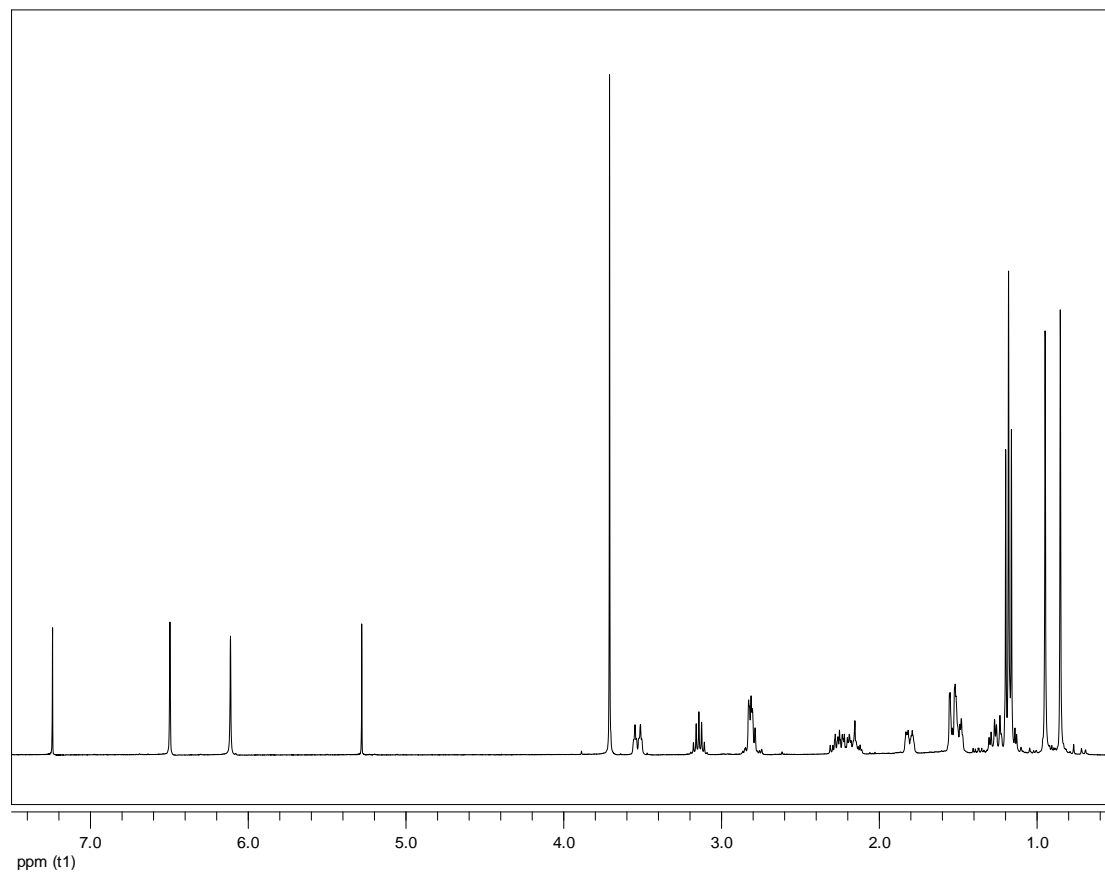

Supplement: Additional file 5: Figure S6. — 1H-NMR spectrum of 12-methoxycarnosic acid (compound 4). (PDF 111 kb) [file 12864_2015_2147_MOESM5_ESM.pdf]

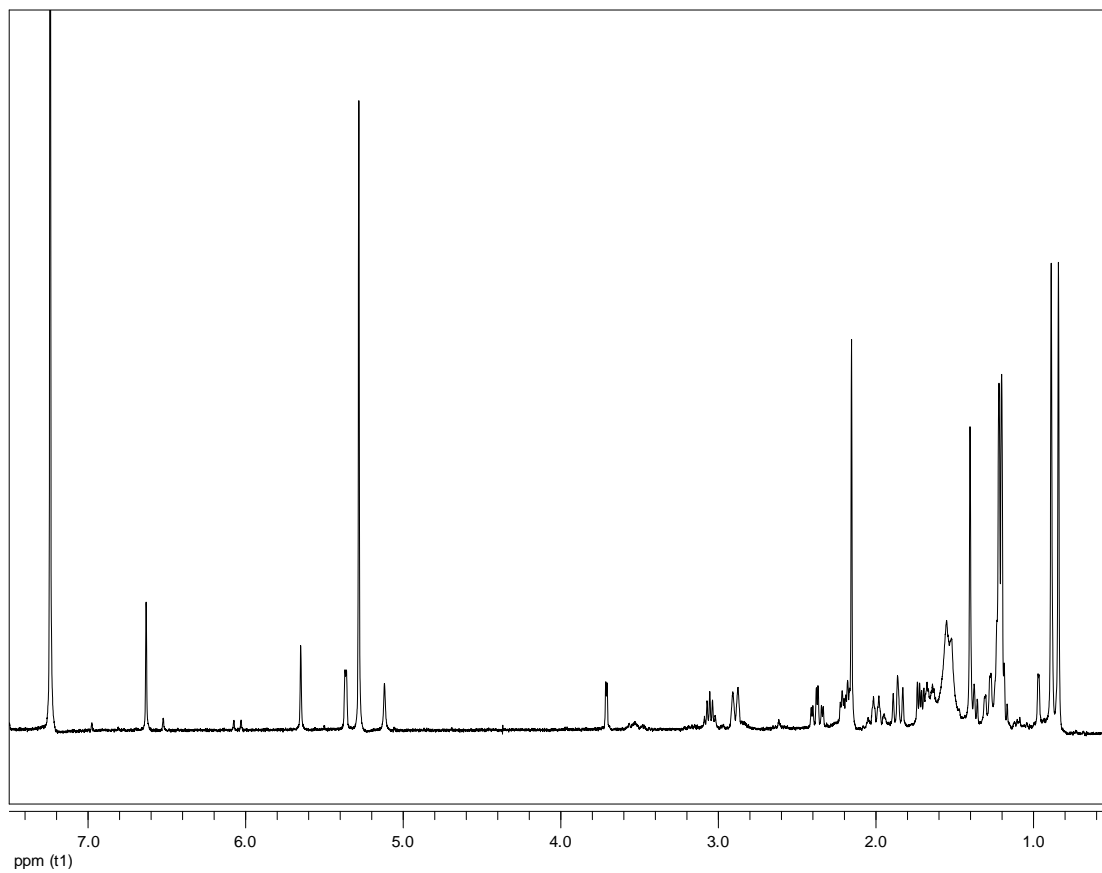

Supplement: Additional file 6: Figure S7. — 1H-NMR spectrum of carnosol (compound 5). (PDF 116 kb) [file 12864_2015_2147_MOESM6_ESM.pdf]

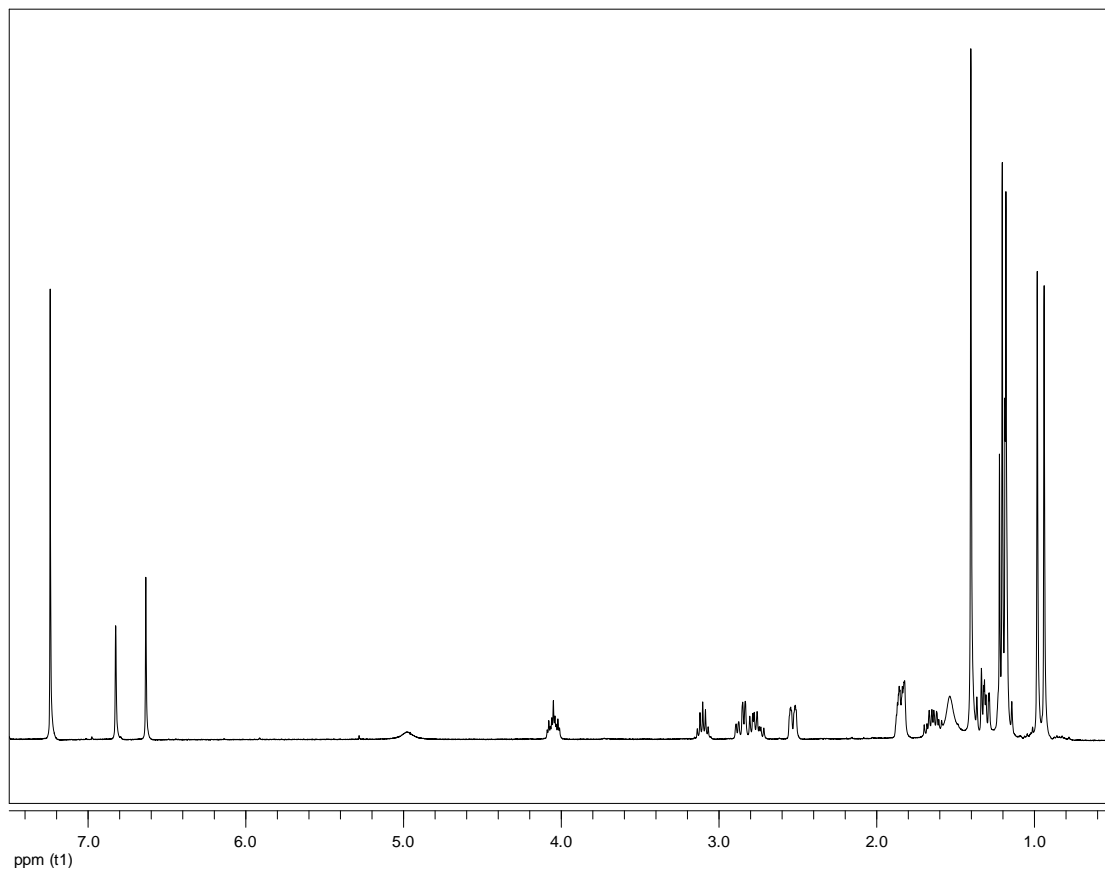

Supplement: Additional file 7: Figure S8. — 1H-NMR spectrum of salviol (compound 6). (PDF 113 kb) [file 12864_2015_2147_MOESM7_ESM.pdf]

A

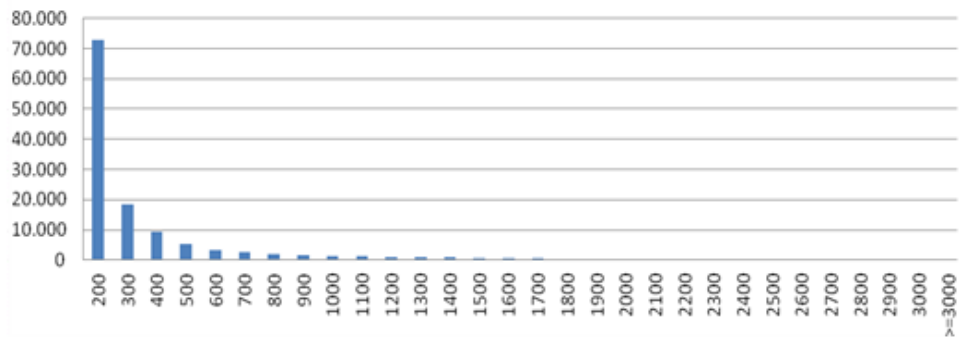

B

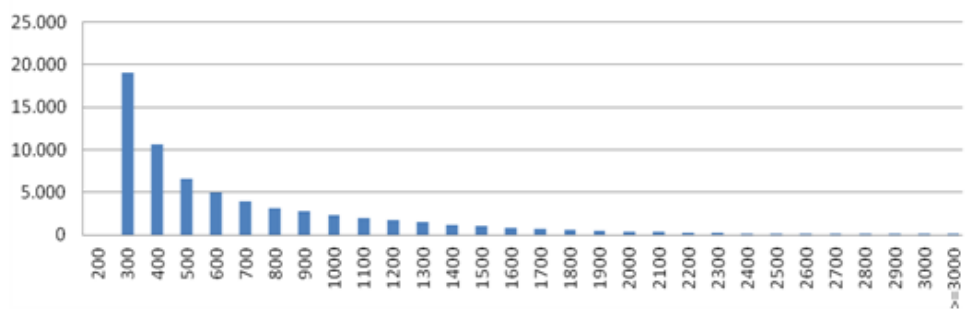

Supplement: Additional file 8: Figure S1. — (A) Distribution of S. pomifera contigs according to their length and (Β) Distribution of S. pomifera clusters of contigs and unigenes according to their length. (PDF 111 kb) [file 12864_2015_2147_MOESM8_ESM.pdf]
