# Supplementary material for: Combined metabolome and transcriptome profiling provides new insights into diterpene biosynthesis in S. pomifera glandular trichomes
Source: BMC Genomics. 2015 Nov 14;16:935. doi: 10.1186/s12864-015-2147-3 (PMC4647624; doi:10.1186/s12864-015-2147-3)
Supplement: Additional file 9: Table S1. — Thirty-five most expressed genes/contigs in M. spicata trichomes based on their FPKM values produced by the RSEM software. In the last column their top hit is referred, produced by BLAST searches in the NR database. The length of the contigs is sometimes not an integer number due to the fact that one contig may correspond to several transcripts of diverse length. The RSEM software was run on the data produced by the transcriptome sequencing of M. spicata trichomes by Jin et al. [37]. (PDF 103 kb) [file 12864_2015_2147_MOESM9_ESM.pdf]

M. spicata contig	Length	FPKM	Top BLAST NR hit
c60211_g2	757.34	42,008.81	hypothetical protein, partial [MENSP MC-2012]
c51259_g1	6,422.00	24,901.96	hypothetical protein MTR_5g050970 [MEDTR]
c59767_g1	647.64	20,343.79	hypothetical protein ppa013682mg [PRUPE]
c53233_g1	1,921.00	10,351.68	RecName: Full=Cytochrome P450 71D18; AltName: Full=(-)-(4S)-Limonene-6-hydroxylase [MENGR]
c71666_g1	397.00	6,539.42	no hit
c40316_g1	384.00	6,162.27	hypothetical protein, partial [MENSP MC-2012]
c63826_g1	972.63	5,910.47	hypothetical protein mgv1a012999mg [ERYGU]
c94072_g1	532.00	5,348.88	RecName: Full=Cytochrome P450 71D94 [MENGR]
c61069_g1	2,035.00	5,178.67	(-)-limonene 3-hydroxylase-like CYP450-dependent oxygenase [MENSP]
c64299_g1	1,496.98	4,682.90	hypothetical protein MIMGU_mgv1a020040mg, partial [ERYGU]
c62984_g1	2,060.82	4,642.08	4S-limonene synthase [MENSP]
c57278_g1	680.00	4,559.92	no hit
**c60448_g1**	**896.80**	**4,088.72**	**PREDICTED: ribulose bisphosphate carboxylase small chain, chloroplastic-like [ERYGU]**
**c61167_g3**	**1,176.35**	**3,722.43**	**PREDICTED: chlorophyll a-b binding protein of LHCII type 1 [CUCME]**
c63697_g2	798.19	3,594.53	hypothetical protein MIMGU_mgv1a016108mg [ERYGU]
c6798_g1	320.00	3,529.31	no hit
c64305_g2	1,465.53	3,439.35	RecName: Full=8-hydroxyquercetin 8-O-methyltransferase; AltName: Full=Flavonol 8-O-methyltransferase [Mentha x piperita]

c80200_g1	686.00	3,385.59	hypothetical protein MIMGU_mgv1a015414mg [ERYGU]
c53657_g3	1,672.00	3,209.08	hypothetical protein MIMGU_mgv1a007849mg [ERYGU]
c59286_g1	938.52	2,923.70	Cu/Zn superoxide dismutase family protein [POPTR]
c61908_g2	844.07	2,876.72	no hit
c59923_g1	1,583.00	2,840.71	PREDICTED: uncharacterized protein LOC104228662 [NICSI]
c63072_g1	1,769.62	2,819.34	hypothetical protein MIMGU_mgv1a023994mg [ERYGU]
c55645_g1	1,936.00	2,756.41	terpene synthase 3 [ORIVU]
c59941_g1	565.06	2,754.35	(E)-beta farnesene synthase [MENAR]
c47337_g1	809.00	2,744.03	no hit
c60831_g1	1,163.00	2,637.39	hypothetical protein JCGZ_03321 [JATCU]
c65365_g1	2,813.00	2,529.49	hypothetical protein JCGZ_23012 [JATCU]
c62174_g1	865.05	2,415.77	hypothetical protein MIMGU_mgv1a009314mg [ERYGU]
c64692_g1	1,454.00	2,354.71	flavonoid 7-O-methyltransferase [Mentha x piperita]
c50263_g1	761.00	2,063.20	PREDICTED: axial regulator YABBY 5-like isoform X1 [NICSI]
c46264_g1	309.00	2,056.55	hypothetical protein MIMGU_mgv11b0121611mg, partial [ERYGU]
c53820_g1	821.45	1,943.53	Ubiquitin supergroup,Ribosomal protein L40e [THECC]
c69178_g1	526.68	1,905.85	elongation factor alpha5, partial [MAIZE]
c63283_g1	1,043.00	1,850.43	ferredoxin-NADP(+) reductase, partial [OCIBA]
